# Comparative analysis of fatty acid profiles across omnivorous, flexitarians, vegetarians, and vegans: insights from the NuEva study

**DOI:** 10.1186/s12944-025-02517-6

**Published:** 2025-04-09

**Authors:** Lea Klein, Claudia Lenz, Karsten Krüger, Stefan Lorkowski, Kristin Kipp, Christine Dawczynski

**Affiliations:** 1 Junior Research Group Nutritional Concepts, Institute of Nutritional Sciences, Friedrich Schiller University Jena, 07743 Jena, Germany; 2 Institute for Sports Science, Department of Exercise Physiology and Sports Therapy, University of Giessen, 35394 Giessen, Germany; 3 Competence Cluster for Nutrition and Cardiovascular Health (nutriCARD) Halle-Jena-Leipzig, 07743 Jena, Germany; 4 Institute of Nutritional Sciences, Friedrich Schiller University Jena, 07743 Jena, Germany; 5 Department for Pediatrics, University Hospital Jena, Jena, Germany

**Keywords:** SFA, TFA, MUFA, PUFA, *n*-3 fatty acid, *n*-6 fatty acid, Fatty acid profile, Inflammation, Plant-based diet

## Abstract

**Background:**

Different dietary choices can influence blood fatty acid profiles, which are crucial for maintaining physiological health and reducing disease risk. In particular, the exclusion of animal foods in vegetarian diets is associated with a higher risk of undersupply of long-chain omega (*n*)-3 fatty acids, which could, potentially, have a negative effect on inflammation. This study aimed to examine differences in plasma and erythrocyte fatty acid profiles as well as inflammation-related biomarkers between various plant-based diets and a regular omnivores diet.

**Methods:**

The Nutritional Evaluation (NuEva) study is a is a parallel-designed trial. Here screening data was used to investigate differences in plasma and erythrocyte fatty acid profiles across omnivores (Western diet; *n* = 62), flexitarians (*n* = 69), vegetarians (*n* = 64) and vegans (*n* = 57). Furthermore, markers associated with inflammation are investigated and correlated with selected fatty acids.

**Results:**

Flexitarians showed lower erythrocyte saturated fatty acids (SFA) than omnivores, while vegans had the lowest plasma SFA. Vegans had higher erythrocyte monounsaturated fatty acids proportions, like oleic acid, than flexitarians and vegetarians. *n*-6 fatty acids, particularly linoleic acid, were highest in vegans and vegetarians. Conversely, omnivores had higher arachidonic acid in erythrocytes. Vegans had lower *n*-3 fatty acids in both plasma and erythrocytes, also reflected in a lower *n*-3 index (eicosapentaenoic acid (EPA) + docosahexaenoic acid (DHA)) values, indicating a trend with restriction of animal foods: omnivores/flexitarians > vegetarians > vegans. While interleukin (IL)-6, IL-8, IL-10, tumor necrosis factor (TNF)-α and high-sensitive C-reactive protein (hsCRP) did not differ between groups, and vegans had lower leptin levels compared to omnivores.

**Conclusions:**

The NuEva study revealed significant impact of dietary patterns on fatty acid profiles, with vegans and vegetarians displaying lower concentrations of SFA and *n*-3 fatty acids, including EPA and DHA, compared to omnivores and flexitarians. Despite the clear differences in fatty acid profiles across the diets, the inflammatory markers measured in our healthy collective are comparable.

**Trial registration:**

Registered under ClinicalTrials.gov Identifier no. NCT03582020.

**Supplementary Information:**

The online version contains supplementary material available at 10.1186/s12944-025-02517-6.

## Introduction

Fatty acids are critical components of human health, serving as key sources of energy, structural components for cell membranes, and modulators of metabolic and signaling pathways [1]. They play a pivotal role in influencing the risk and progression of non-communicable diseases, e.g. cardiovascular disease [1]. Many fatty acids can be synthesized endogenously; however, the omega (*n*)-3 and *n*-6 polyunsaturated fatty acids (PUFA) α-linolenic acid (ALA; C18:3*n*-3) and linoleic acid (LA; C18:2*n*-6) are essential and must be provided in the diet [2]. These fatty acids compete for the same rate-limiting desaturase enzymes for their conversion into long-chain (LC) PUFA like arachidonic acid (ARA; C20:4*n*-6), eicosapentaenoic acid (EPA; C20:5*n*-3), docosapentaenoic acid (DPA; C22:5*n-*3), and docosahexaenoic acid (DHA; C22:6*n*-3) [3]. While ARA is a precursor of eicosanoids like prostaglandins and leukotrienes, that promote inflammation, mediators derived from LC *n*-3 PUFA are known for their anti-inflammatory properties [4]. More recent research suggests associations between chronic inflammation and an increased risk of various chronic diseases. Indeed, elevated levels of inflammatory markers such as high-sensitivity C-reactive protein (hsCRP), interleukin (IL)-6 or tumor necrosis factor (TNF)-α have been found to be associated with pathogenetic mechanisms of numerous chronic diseases such as type 2 diabetes mellitus [5] and cardiovascular disease (CVD) [6]. In contrast, concentrations of adiponectin were inversely related to these diseases [7, 8]. The balance of fatty acids in the diet may therefore influence chronic inflammation in the body.

Dietary choices mainly influence the abundance and relation of circulating fatty acids in the human body. Omnivorous diets typically include higher amounts of saturated fatty acids (SFA), while vegetarian and vegan diets are characterized by higher intakes of PUFA [9–11]. This shift towards PUFA consumption in place of SFA is associated with a reduced risk of coronary heart disease [12]. However, it is important to note that vegetarians and vegans typically have low or negligible intakes of EPA, DPA and DHA, due to dietary restrictions of fish and meat [13]. Although the body can convert ALA to EPA, DPA and DHA, this conversion is limited [3] and can be further reduced by simultaneously high intake of LA [14]. This *n*-6 PUFA is abundant in a plant-based diet as it is an important component of vegetable oils, margarine, avocados and nuts. Consequently, vegetarians and especially vegans may be at risk for a suboptimal *n*-6/*n*-3 ratio and LC *n*-3 PUFA status, which in turn may negatively affect inflammatory responses.

To comprehensively assess the benefits and risks associated with a vegetarian and vegan diet the Nutritional Evaluation (NuEva) study was conducted. This study was designed to investigate the impact of dietary patterns differing in their proportion of animal foods on nutrient status and cardiovascular risk factors [15]. The investigation included four dietary patterns: traditional Western diet (omnivores), flexitarians, vegetarians, and vegans. Based on the baseline data, practical nutritional concepts, including energy- and nutrient-optimized menu plans were developed for each dietary pattern to mitigate potential nutrient deficits. Participants should adhere to these menu plans for 12 months. The present data focuses on the screening (baseline) fatty acid profiles in the plasma and erythrocyte lipids, reflecting short-term (2–3 days) and mid-term (approx. 120 days) fatty acid intake, respectively. Furthermore, markers associated with inflammation were investigated and correlated with selected fatty acids, providing insights into the physiological consequences of these dietary patterns.

## Materials and Methods

### Participants

**Fig. 1:**
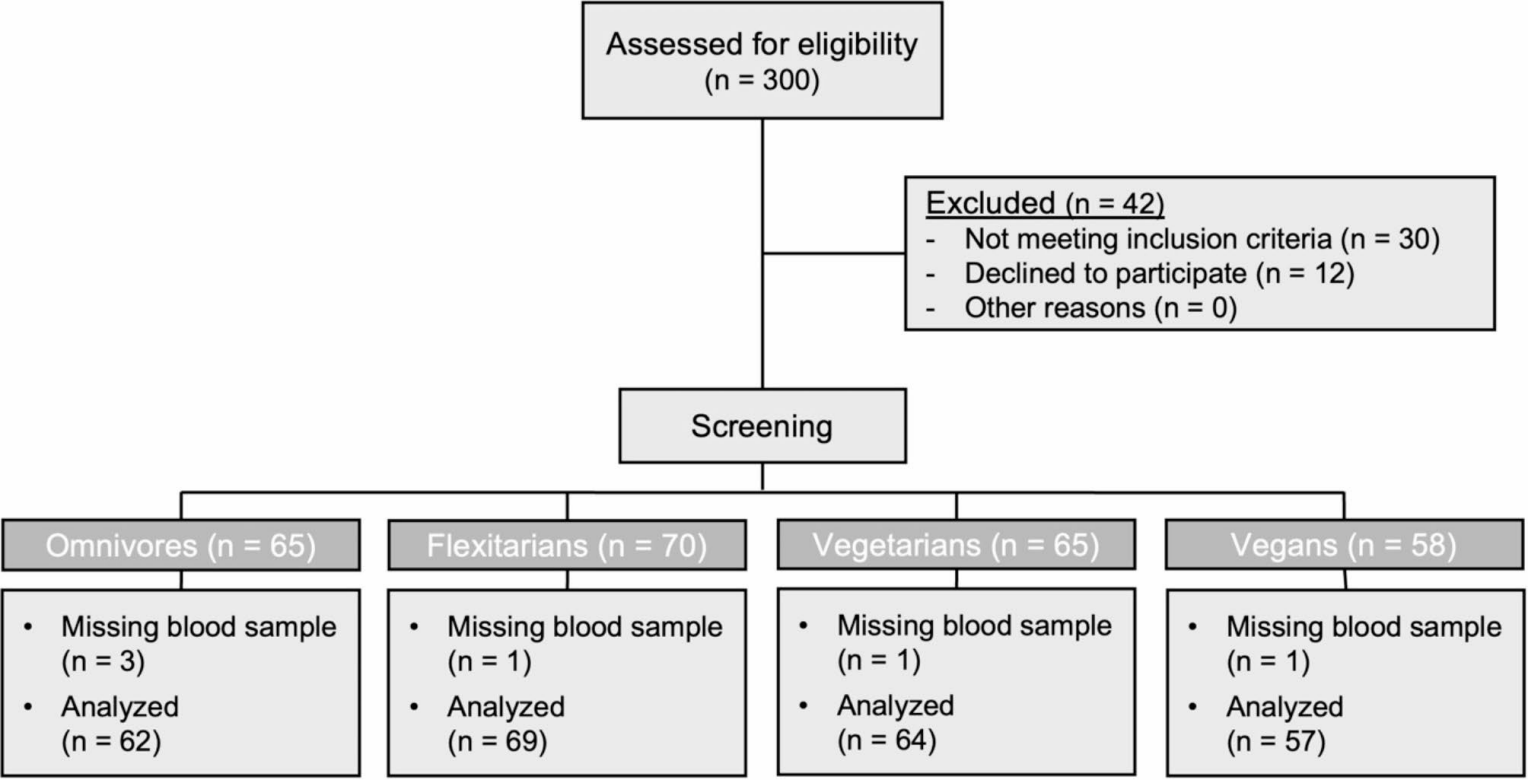
Flow diagram of the selection of NuEva study participants. A total of 300 subjects were enrolled in the study. 42 subjects were excluded from the study, either because they did not meet the inclusion criteria or because they declined to participate. The participants were divided into four groups based on their dietary habits: omnivores, flexitarians, vegetarians, and vegans

In the summer and autumn of 2018, healthy adults aged 18 to under 70 years, who had been adhering to an omnivorous, flexitarian, vegetarian, or vegan diet for a minimum of one year prior to their participation in the study, were recruited. Their adherence to these dietary patterns was verified through an interview prior to enrollment and confirmed by a dietary protocol before the initial study visit. The omnivorous diet resembled a Western diet, characterized by daily meat and sausage consumption, whereas the flexitarian diet included meat and sausages only occasionally (1–2 times per week). Both groups had unrestricted, though not mandatory, fish consumption. Vegetarians were defined as individuals consuming dairy products and eggs in addition to plant-based foods, while vegans were those consuming only plant-derived foods. Additional exclusion criteria for the study have been detailed elsewhere [15]. In this analysis, 62 omnivores, 69 flexitarians, 64 vegetarians, and 57 vegans who completed blood sampling at the screening (baseline) were included (Fig. 1).

### Study design and diets

The NuEva study commenced with a run-in period to document and evaluate the dietary habits of participants through a five-day dietary record. To assess the nutritional and health status of the participants, a single blood draw along with the collection of fecal and 24-hour urine samples were conducted prior to the initiation of the study during the screening phase. Throughout the 12-month intervention phase, participants underwent checkups every three months, which included anthropometric measurements, pulse, blood pressure assessments, and blood sampling. In addition, participants received daily menu plans optimized for nutrient intake. Detailed information on the study design can be found in the published study protocol [15].

The study protocol was reviewed and approved by the Ethical Committee of the Friedrich Schiller University Jena (number: 5504-03/18). The NuEva study was registered before launching (Clinical-Trials.gov Identifier: NCT03582020).

### Nutrient intake and body composition

Participants were required to provide a self-reported dietary intake over a period of five days. This dietary record utilized the “*Freiburger Ernährungsprotokoll*” template, which included a wide range of common foods and typical portion sizes. For the NuEva study, the template was modified to include additional foods that are commonly consumed in vegetarian and vegan diets, such as tofu, vegan yogurt alternatives, plant-based drinks, soy products, seitan, tempeh, maple syrup, and agave syrup. Participants also had the option to add further foods that were not originally listed. If a food item was not available in the PRODI database, it was manually entered, and the nutritional information was sourced directly from the packaging, including details on any fortifications, such as vitamin B_12_ or calcium. The daily energy and nutrient intake were calculated using the PRODI^®^ software (version 6.4, Nutri-Science, Stuttgart, Germany).

In the screening phase, body composition was assessed utilizing a Body Impedance Analyzer (seca 515/514, seca, Hamburg, Germany).

### Laboratory Parameters

#### Sample Collection

Blood samples were collected by venipuncture after at least 12 h overnight fasting. For separation of plasma, blood was collected in lithium-heparin monovettes, fully coagulated at room temperature, and centrifuged for 10 min (2762 x *g*, 4 °C). After plasma was removed, the residual erythrocytes were washed with an equal volume of physiological sodium chloride solution (0.9%) and centrifuged for 10 min (1300 x *g*, 4 °C). This step was repeated until the supernatant was clear (2 x). All samples were stored at − 80 °C until analysis.

#### Lipid Extraction and Fatty Acid Analysis

Plasma and erythrocyte lipids were extracted according to the method of Folch and Bligh and Dyer et al. [16, 17] using a methanol/chloroform/water mixture (2:1:1, v/v/v). The extracted lipids were saponified and methylated with NaOCH_3_ (Carl Roth, Karlsruhe, Germany) and BF_3_ (Sigma-Aldrich, Steinheim, Germany) as described before [18]. For purification of the obtained products after methylation, thin-layer chromatography on silica gel aluminum plates (Merck KGaA, Darmstadt, Germany) with hexane: diethyl ether: acetic acid (85:15:0.2, v/v/v) was used. Fatty acid methyl esters (FAME) were measured via a gas chromatograph (GC-17 V3; Shimadzu, Duisburg, Germany) with a flame ionization detector. Different standards were used for the identification of fatty acid peaks (No. 463, 674 (NU-CHEK PREP; INC., U.S), BR2, BR4, and ME 93 (Larodan Fine Chemicals, Solna, Sweden), Menhaden (Sigma Aldrich, Steinheim, Germany). Quantification of each FAME was performed by solution software (LabSolution LC/GC release 5.92, Shimadzu). The outputs of each fatty acid concentration were given as the percentage of the total area of total fatty acid methyl esters (%FAME).

For calculations of the sum of *n*-3 PUFA, the following *n*-3 PUFA were included: ALA (C18:3*n*-3), eicosatetraenoic acid (C20:4*n*-3), EPA (C20:5*n*-3), DPA (C22:5*n*-3) and DHA (C22:6*n*-3). The sum of *n*-6 PUFA comprises following fatty acids: LA (C18:2*n*-6), γlinolenic acid (C18:3*n*-6), C20:2*n*-6, dihomoγlinolenic acid (C20:3*n*-6), ARA (C20:4*n*-6), C22:4*n*-6 and C-22:5*n*-6. The *n*-3 index was calculated by using the sum of EPA and DHA in erythrocyte lipids. The sum of LC *n*-3 fatty acids comprises EPA, DPA, and DHA. Further, %*n-*6 and %*n*-3 in highly unsaturated fatty acids (HUFA), which comprise fatty acids with three or more double bonds in their carbon chains, were calculated according to formula (1)) and (2), respectively.

1 $$\eqalign{&\% n-6\:in\:HUFA:100\:\times \cr & \frac{{C20:3n-6+ARA + C22:4n-6\:+C22:5n-6}}{{C20:3n-6\:+ARA + C22:4n-6+C22:5n-6 + C20:3n-3+C20:4n-3\:+EPA\:+DPA\:+DHA}} \cr} $$

2 $$\eqalign{&\% n{\text{ }}-3\:in\:HUFA:100\:\times\cr & \frac{{C20:3n-3+C20:4n-3\: + EPA + DPA\: + DHA}}{{C20:3n-6\: + ARA+22:4n{\text{ }}6+\:C22:5n-6 +\:C20:3n -3 + C20:4n-3\: +EPA\:+DPA\:+ DHA}} \cr} $$

### Measurement of inflammatory biomarkers

hsCRP was measured in plasma at the Institute of Clinical Chemistry and Laboratory Diagnostics, University Hospital Jena, using a Cobas 8000 (Roche, Mannheim, Germany). Plasma samples were also used for determination of IL-6, IL-8, IL-10 and TNF-α, leptin and adiponectin by Luminex Multiplex Assay/Luminex Assay (Luminex LX200, Biotechne, Minneaplois, USA) according to the manufacturer’s recommendations.

### Statistical analyses

The analysis of fatty acids and inflammation-related biomarkers was an exploratory aim. The sample size was therefore planned for the main parameters (blood lipids) of the trial. The power calculation was conducted for the LDL cholesterol/HDL cholesterol ratio, based on data from Li et al. [19]. With a sample size of 44 participants per group, an 80% power was achieved. Anticipating a 25% drop-out rate, we aimed to recruit a minimum of 55 participants per group. The power analysis for the NuEva study was conducted using G*Power (The G*Power Team, Düsseldorf, Germany, version 3.1.9.2). The statistical analysis was performed by SPSS statistics (IBM, Chicago, USA, version 29.0.0.0). If the data of the four groups followed a normal distribution (tested with Shapiro-Wilk), ordinary one-way ANOVA (using Benjamini-Hochberg correction) was applied for non-paired samples. Otherwise, Kruskal-Wallis test (using Benjamini-Hochberg correction) was conducted. Normally distributed data were presented as mean ± standard deviation, whereas skewed data were presented as median/interquartile range (IQR). Correlation analyses were performed based on Spearman’s rank correlation. Statistical significance is referred to *p*-values smaller than 0.05. The violin plots were generated using R studio (Public Benefit Corporation, Boston, Massachusetts, USA, version 2022.12.0) running R software (The R Foundation for Statistical Computing, Vienna, Austria, version 4.2.3).

## Results

### Distribution of Plasma Fatty Acids

The sum of SFA in plasma was lower in vegans compared to omnivores, flexitarians and vegetarians (*p* < 0.001). This is mainly due to reduced levels of palmitic acid (C16:0) in vegans compared to all groups (*p* < 0.001), which accounts for the largest concentration of SFA in plasma. For myristic (C14:0) acid and pentadecanoic acid (C15:0), the highest concentrations were observed in flexitarians and vegetarians, which differed significantly from omnivores (*p* = 0.03) and vegans (*p* < 0.001), while the lower amount in vegans also differed from omnivores (*p* < 0.001). Furthermore, the concentration of heptadecanoic acid (C17:0) was equal in omnivores, flexitarians, and vegetarians, but significantly lower in vegans (*p* ≤ 0.001), while omnivores had higher amounts of stearic acid compared to flexitarians, vegetarians and vegans (*p* ≤ 0.001). In contrast, arachidic acid (C20:0) was the only SFA with a higher concentration in vegans compared to all groups (*p* < 0.001) and moreover vegetarians showed higher values than flexitarians (*p* < 0.001; Table 1).

**Table 1:** Fatty acid profiles (%FAME) in plasma lipids according to diet group

	sex	WD (23 m, 39 w)		Flex (14 m, 55 w)		VG (18 m, 46 w)		VN (17 m, 40 w)	
**SFA**									
C14:0	all	0.65 / 0.40	a	0.78 / 0.35	b	0.84 / 0.48	b	0.49 / 0.23	c
C15:0	all	0.20 / 0.08	a	0.23 / 0.06	b	0.24 / 0.09	b	0.10 / 0.04	c
C16:0	all	21.56 / 2.62	a	22.14 / 3.07	a	21.38 / 3.06	a	18.74 / 2.29	b
C17:0	all	0.23 / 0.05	a	0.23 / 0.04	a	0.23 / 0.05	a	0.17 / 0.04	b
C18:0	all	7.00 / 0.98	a	6.41 / 1.00	b	6.59 / 1.11	b	6.63 / 0.92	b
C20:0	all	0.04 / 0.02	a, b	0.04 / 0.01	a	0.04 / 0.02	b	0.06 / 0.02	c
Σ *SFA*	all	30.66 / 2.61	a	30.57 / 3.35	a	30.16 / 3.00	a	26.91 / 2.35	b
**MUFA**									
C16:1n-7	all	1.57 / 0.69	a	1.64 / 0.87	a	1.68 / 0.60	a	1.09 / 0.63	b
C18:1*n*-9	all	21.95 / 3.59	a	21.48 / 3.98	a	21.77 / 3.52	a	23.00 / 3.63	a
C20:1*n*-9	all	0.16 / 0.05	a	0.14 / 0.05	a	0.16 / 0.05	a	0.20 / 0.06	b
Σ *MUFA*	all	25.08 / 3.83	a	24.88 / 4.04	a	25.26 / 3.76	a	25.96 / 4.53	a
***n*** **-6 PUFA**									
C18:2*n*-6	m w all	28.72 / 5.62 29.56 / 4.04 29.20 / 4.92	a a a	31.75 / 10.36 30.66 / 4.55 30.66 / 5.67	a, b a, b b	31.99 / 2.83 30.99 / 4.58 31.52 / 4.49	b b b	37.80 / 7.43 35.08 / 4.87 35.57 / 6.04	c c c
C18:3*n*-6	all	0.34 / 0.22	a	0.29 / 0.14	a	0.33 / 0.14	a	0.31 / 0.13	a
C20:2*n*-6	all	0.16 / 0.06	a	0.15 / 0.05	b	0.16 / 0.05	a, b	0.22 / 0.07	c
C20:3*n*-6	all	1.35 / 0.38	a	1.32 / 0.49	a	1.46 / 0.49	a	1.30 / 0.53	a
C20:4*n*-6	m w all	6.99 / 1.32 6.39 / 2.07 6.57 / 1.82	a a a	5.26 / 2.43 5.82 / 1.64 5.43 / 1.80	b b b	4.90 / 1.19 5.68 / 1.42 5.34 / 1.57	b b b	4.70 / 2.87 5.21 / 1.69 5.20 / 1.79	b b b
C22:4*n*-6	all	0.17 / 0.09	a	0.17 / 0.05	a	0.18 / 0.07	a	0.16 / 0.05	a
C22:5*n*-6	all	0.13 / 0.07	a	0.10 / 0.06	b	0.10 / 0.05	b	0.09 / 0.07	b
Σ *n-6 PUFA*	all	38.42 / 5.11	a	38.20 / 4.62	a	39.21 / 5.03	a	42.77 / 4.84	b
***n*** **-3 PUFA**									
C18:3*n*-3	m w all	0.82 ± 0.31 0.86 / 0.44 0.78 / 0.41	a, c a a	0.51 ± 0.46 0.51 / 0.19 0.51 / 0.20	b b b	0.66 ± 0.60 0.48 / 0.19 0.50 / 0.22	a, b b b	1.04 ± 0.86 0.72 / 0.37 0.80 / 0.38	c a a
C20:4*n*-3	all	0.08 / 0.07	a	0.06 / 0.04	a, b	0.07 / 0.05	a, b	0.06 / 0.06	b
C20:5*n*-3	m w all	0.72 / 0.43 0.60 / 0.41 0.63 / 0.38	a a a	0.42 / 0.28 0.43 / 0.29 0.43 / 0.28	b a b	0.50 / 0.33 0.39 / 0.27 0.42 / 0.33	a, b b b	0.24 / 0.29 0.30 / 0.14 0.30 / 0.23	b b c
C22:5*n*-3	all	0.41 / 0.13	a	0.32 / 0.11	b	0.34 / 0.13	b	0.35 / 0.13	b
C22:6*n*-3	m w all	1.34 / 0.48 1.42 / 0.60 1.40 / 0.57	a a a	0.99 / 0.48 1.13 / 0.46 1.11 / 0.39	a, b b b	0.84 / 0.58 0.82 / 0.44 0.84 / 0.48	b c c	0.70 / 0.52 0.92 / 0.34 0.89 / 0.38	b c c
*n*-3 LC PUFA	all	2.46 / 0.73	a	1.93 / 0.69	a, b	1.65 / 0.83	b, c	1.57 / 0.54	c
Σ *n-3 PUFA*	all	3.50 / 1.16	a	2.59 / 0.83	b	2.37 / 0.98	b	2.42 / 0.77	b
Σ *PUFA*	all	42.04 / 4.37	a	41.47 / 5.39	a	41.43 / 5.50	a	45.78 / 4.60	b
%*n*-6 HUFA^1^	all	76.81 / 6.44	a	78.97 / 6.04	b	81.49 / 5.94	c	81.77 / 6.04	c
%*n*-3 HUFA^2^	all	23.49 / 6.46	a	21.32 / 5.46	b	18.73 / 6.49	c	18.34 / 6.94	c
**TFA**									
C16:1t9	all	0.32 / 0.08	a	0.34 / 0.08	a, b	0.33 / 0.08	a, b	0.36 / 0.13	b
C18:1t9	all	0.14 / 0.05	a	0.11 / 0.04	b	0.10 / 0.04	b	0.06 / 0.04	c
Σ *TFA*	all	0.93 / 0.29	a	0.93 / 0.19	a	0.88 / 0.24	a	0.62 / 0.18	b
**CLA**		0.13 / 0.05	a	0.12 / 0.17	b	0.09 / 0.19	b	0.05 / 0.06	c
**Ratios**									
*n*-6/*n*-3	all	11.15 / 3.45	a	14.62 / 4.65	b	16.30 / 2.27	b, c	18.86 / 6.78	c
ARA/EPA	all	10.34 / 6.47	a	12.50 / 5.35	b	12.97 / 8.75	b	17.44 / 10.71	c
ARA/DHA	all	4.55 / 2.22	a	5.08 / 1.83	a	5.96 / 3.08	b	5.92 / 2.23	b
LA/ALA	all	36.15 / 18.17	a	58.93 / 20.21	b	61.72 / 25.96	b	44.91 / 25.87	c
ARA/LA	all	0.23 / 0.09	a	0.18 / 0.07	b	0.16 / 0.06	b	0.14 / 0.05	c

Results are presented as median / IQR. Diet groups that do not share indices (a, b,c) differ significantly (Kruskal-Wallis test followed by Benjamini-Hochberg correction (*p* < 0.05)). Abbreviations: WD = omnivores, Flex = flexitarians, VG = vegetarians, VN = vegans, m = men, w = women, SFA = saturated fatty acids, MUFA = monounsaturated fatty acids, PUFA = polyunsaturated fatty acids, *n =* omega, LC = long-chain, HUFA = highly unsaturated fatty acids, TFA = trans fatty acids, CLA = conjugated linoleic acids, ARA = arachidonic acid, EPA = eicosapentaenoic acid, DHA = docosahexaenoic acid, LA = linoleic acid, ALA = α-linolenic acid

^1^Percentage of *n*-6 PUFA in fatty acids with three or more double bonds

^2^Percentage of *n*-3 PUFA in fatty acids with three or more double bonds

**Table 2:** Fatty acid profiles (%FAME) in erythrocyte lipids according to diet group

	sex	WD (23 m, 39 w)		Flex (14 m, 55 w)		VG (18 m, 46 w)		VN (17 m, 40 w)	
SFA									
C14:0	all	0.23 / 0.11	a	0.25 / 0.09	a	0.26 / 0.10	a	0.18 / 0.10	b
C15:0	all	0.15 / 0.05	a	0.16 / 0.04	a	0.16 / 0.04	a	0.08 / 0.04	b
C16:0	all	23.28 / 2.47	a	22.86 / 2.67	a	22.58 / 2.72	a	22.20 / 3.06	a
C17:0	all	0.27 / 0.04	a	0.26 / 0.04	a	0.27 / 0.06	a	0.22 / 0.06	b
C18:0	all	11.96 / 2.26	a, b	11.18 / 3.59	a	11.91 / 2.43	a, b	12.56 / 3.81	b
C20:0	all	0.07 / 0.02	a	0.08 / 0.03	b	0.08 / 0.03	a, b	0.10 / 0.04	c
Σ *SFA*	all	38.46 / 2.43	a	37.61 / 2.09	b	38.00 / 2.03	a, b	37.62 / 2.19	a, b
**MUFA**									
C16:1*n*-7	all	0.27 / 0.12	a	0.25 / 0.14	a	0.26 / 0.13	a	0.19 / 0.12	b
C18:1*n*-9	all	15.89 / 2.18	a, b	15.68 / 2.21	a	15.72 / 1.39	a	16.39 / 2.17	b
C20:1*n*-9	all	0.33 / 0.18	a	0.24 / 0.11	b	0.30 / 0.17	a	0.41 / 0.23	c
C24:1*n*-9	all	0.10 / 0.07	a	0.11 / 0.07	a	0.11 / 0.06	a	0.12 / 0.07	a
Σ *MUFA*	all	17.89 / 2.26	a, b	17.49 / 2.22	a	17.64 / 1.50	a	18.27 / 2.61	b
***n*** **-6 PUFA**									
C18:2*n*-6	m w all	11.81 / 1.45 11.61 / 2.40 11.74 / 1.79	a a a	12.78 / 3.63 12.00 / 1.74 12.15 / 2.03	a a a, b	12.39 / 3.22 12.98 / 2.82 12.76 / 2.94	a b b	14.45 / 2.20 13.47 / 2.74 13.84 / 2.40	b b c
C18:3*n*-6	all	0.04 / 0.02	a	0.05 / 0.02	b	0.05 / 0.02	b	0.04 / 0.02	a
C20:2*n*-6	all	0.21 / 0.04	a	0.22 / 0.05	a	0.23 / 0.07	b	0.34 / 0.13	c
C20:3*n*-6	all	1.34 / 0.34	a	1.38 / 0.37	a	1.53 / 0.40	b	1.51 / 0.52	b
C20:4*n*-6	m w all	14.17 / 1.70 13.86 / 2.38 13.98 / 2.38	a a a	13.73 / 3.40 13.61 / 1.41 13.61 / 1.79	a, b a a, b	13.92 / 3.24 13.42 / 1.39 13.50 / 1.46	a, b a a, b	12.64 / 2.07 13.52 / 2.85 12.93 / 2.66	b a b
C22:2*n*-6	all	0.04 / 0.04	a	0.04 / 0.06	a, b	0.04 / 0.06	a	0.03 / 0.05	b
C22:4*n-*6	all	2.68 / 0.71	a	2.69 / 0.73	a	2.97 / 0.73	b	2.99 / 0.92	b
C22:5*n*-6	all	0.37 / 0.11	a	0.38 / 0.17	a	0.44 / 0.17	b	0.34 / 0.12	a
Σ *n-6 PUFA*	all	30.88 / 2.24	a	30.76 / 2.59	a	31.75 / 2.77	b	32.15 / 2.89	b
***n*** **-3 PUFA**									
C18:3*n*-3	m w all	0.17 ± 0.05 0.17 ± 0.06 0.16 / 0.07	a a a	0.19 ± 0.08 0.18 ± 0.07 0.17 / 0.06	a a a, b	0.20 ± 0.06 0.18 ± 0.06 0.18 / 0.07	a a a, b	0.23 ± 0.12 0.21 ± 0.10 0.19 / 0.08	a a b
C20:3*n*-3	all	0.08 / 0.02	a	0.08 / 0.04	a, b	0.07 / 0.03	b	0.08 / 0.03	a
C20:4*n*-3	all	0.05 / 0.02	a	0.05 / 0.02	a	0.05 / 0.03	a	0.03 / 0.02	b
C20:5*n*-3	m w all	0.68 / 0.39 0.64 / 0.29 0.67 / 0.31	a a a	0.61 / 0.25 0.59 / 0.28 0.59 / 0.28	a a a	0.58 / 0.31 0.46 / 0.31 0.49 / 0.26	a b b	0.36 / 0.23 0.31 / 0.15 0.32 / 0.18	b c c
C22:5*n*-3	all	1.92 / 0.33	a	1.90 / 0.42	a	1.98 / 0.75	a	1.81 / 0.52	a
C22:6*n*-3	m w all	2.89 / 1.42 3.37 / 1.08 3.32 / 1.28	a a a	3.18 / 0.86 3.21 / 1.09 3.21 / 1.09	a a a	2.42 / 1.27 2.68 / 0.95 2.66 / 1.04	a a, b b	2.09 / 1.03 2.35 / 0.80 2.29 / 0.96	b c c
*n*-3 index	all	3.97 / 1.49	a	3.71 / 1.21	a	3.17 / 1.15	b	2.66 / 1.06	c
*n*-3 LC PUFA	all	5.92 / 1.61	a	5.60 / 1.48	a, b	5.43 / 1.21	b	4.47 /1.22	c
Σ *n-*3 PUFA	all	6.26 / 1.58	a	5.93 / 4.59	a, b	5.70 / 1.28	b	4.82 / 1.17	c
Σ PUFA	all	37.33 / 2.49	a	36.76 / 2.37	a	37.37 / 2.87	a	37.23 / 2.51	a
%*n*-6 HUFA^1^	all	75.38 / 6.36	a	75.21 / 5.47	a	77.82 / 4.85	b	80.52 / 4.53	c
%*n*-3 HUFA^2^	all	24.66 / 6.19	a	25.65 / 6.12	a	21.95 / 4.83	b	19.79 / 4.55	c
**TFA**									
C16:1t9	all	0.09 / 0.02	a	0.10 / 0.02	b	0.10 / 0.03	b	0.09 / 0.03	a
C18:1t9	all	0.08 / 0.03	a	0.08 / 0.03	a	0.07 /0.02	a	0.06 / 0.02	b
Σ *TFA*	all	0.40 / 0.12	a	0.41 / 0.12	a	0.41 / 0.12	a	0.27 / 0.10	b
**CLA**	all	0.09 / 0.03	a	0.10 / 0.03	a	0.10 / 0.04	a	0.03 / 0.03	b
**Ratios**									
*n*-6/*n*-3	all	4.70 / 1.55	a	4.76 / 1.31	a	5.67 / 1.68	b	6.72 / 2.12	c
ARA/EPA	all	21.25 / 12.37	a	21.16 / 11.85	a	28.01 / 15.41	b	39.57 / 27.27	b
ARA/DHA	all	4.18 / 1.62	a	4.08 / 1.36	a	5.16 / 1.84	b	5.63 / 1.97	b
LA/ALA	all	75.87 / 31.97	a	72.04 / 21.29	a	72.33 / 21.76	a	72.27 / 34.48	a
ARA/LA	all	1.19 / 0.30	a	1.11 / 0.26	b	1.05 / 0.30	b	0.95 / 0.25	c

Results are presented as median / IQR. Diet groups that do not share indices (a, b,c) differ significantly (Kruskal-Wallis test followed by Benjamini-Hochberg correction (*p* < 0.05)). Abbreviations: WD = omnivores, Flex = flexitarians, VG = vegetarians, VN = vegans, m = men, w = women, SFA = saturated fatty acids, MUFA = monounsaturated fatty acids, PUFA = polyunsaturated fatty acids, *n =* omega, LC = long-chain, HUFA = highly unsaturated fatty acids, TFA = trans fatty acids, CLA = conjugated linoleic acids, ARA = arachidonic acid, EPA = eicosapentaenoic acid, DHA = docosahexaenoic acid, LA = linoleic acid, ALA = α-linolenic acid

^1^Percentage of *n*-6 PUFA in fatty acids with three or more double bonds

^2^ Percentage of *n*-3 PUFA in fatty acids with three or more double bonds

Regarding total monounsaturated fatty acids (MUFA), no differences appeared between the four groups, but alterations in individual MUFA patterns emerged for palmitoleic acid (C16:1*n*-7) and eicosenoic acid (C20:1 *n*-9). While palmitoleic acid was lower in vegans, the level of eicosenoic acid in this group was higher compared to omnivores, flexitarians and vegetarians (both *p* < 0.001). For oleic acid (C18:1*n*-9), no significant differences were found between the groups (Table 1), but a trend for higher oleic acid values in vegans compared to flexitarians (*p* = 0.07) and vegetarians (*p* = 0.08) emerged.

**Fig. 2:**
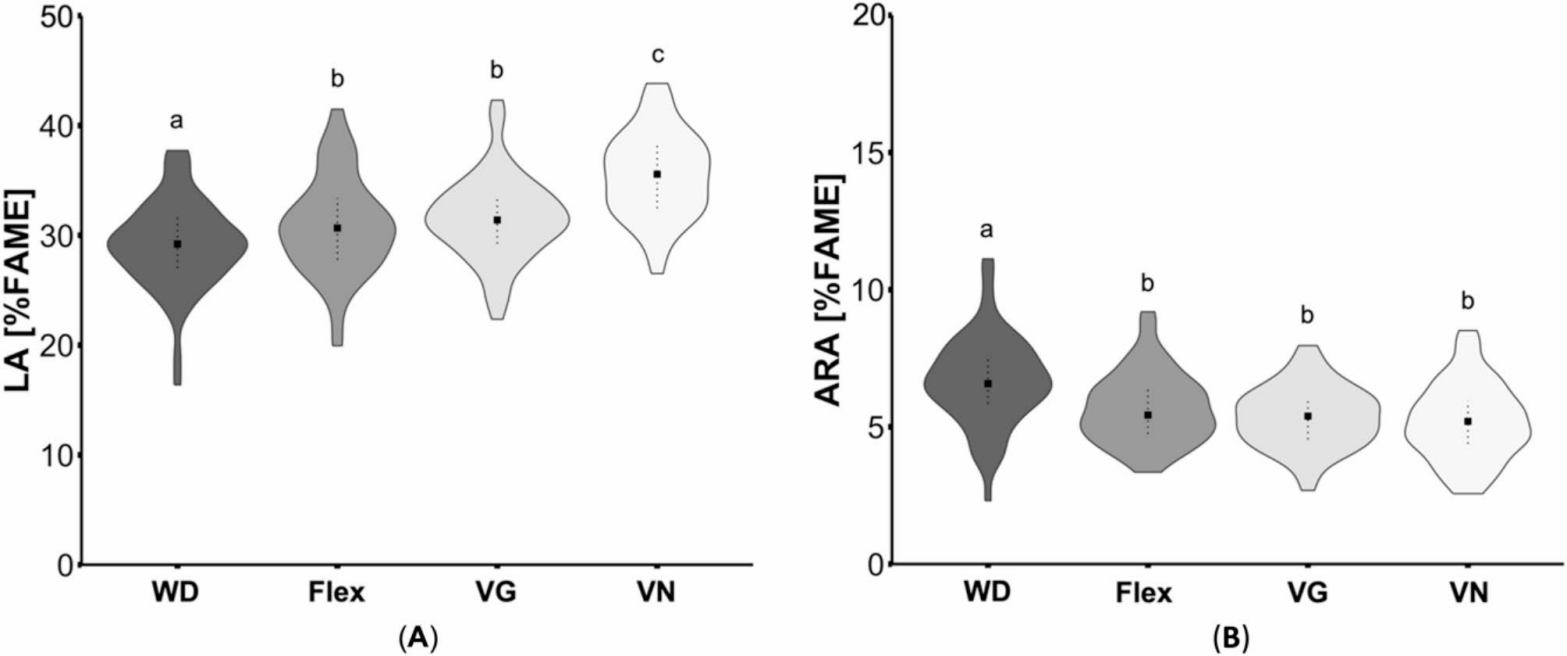
Comparison of selected plasma *n*-6 fatty acids of subjects with different dietary patterns. (**A**) Linoleic acid (LA) and (**B**) arachidonic acid (ARA) concentrations in plasma lipids are presented as the percentage of the total area of total fatty acid methyl esters (%FAME). In total, 62 omnivores (Western diet; WD), 69 flexitarians (Flex), 64 vegetarians (VG) and 57 vegans (VN) were analyzed. The results are shown as violin plots with median (indicated as squares) and 25- and 75-quartile ranges (indicated as dotted lines). Diet groups that do not share indices (a, b,c) differ significantly (Kruskal-Wallis test followed by Benjamini-Hochberg correction (*p* < 0.05))

Overall, the sum of PUFA in plasma was highest in vegans (*p* < 0.001). The vegan group revealed also noticeably higher *n*-6 PUFA in plasma compared to omnivores, flexitarians and vegetarians (*p* < 0.001). Vegans had the highest concentrations of LA (C18:2*n*-6) and eicosadienoic acid (C20:2*n*-6) compared omnivores, flexitarians, and vegetarians (both *p* < 0.001; Fig. 2A). In contrast, omnivores had the lowest amounts for those fatty acids, which were also different from flexitarians (both *p* ≤ 0.01) and vegetarians (LA, *p* < 0.001; Fig. 2A). For ARA (C20:4*n*-6) and docosatetraenoic acid (C22:4*n*-6), the opposite was observed, with omnivores showing slightly higher concentrations than flexitarians (both *p* ≤ 0.01), vegetarians and vegans (both *p* ≤ 0.02; Table 1; Fig. 2B). For the major fatty acids, we analyzed men and women separately, although we generally did not observe significant sex-specific differences. In total, the average concentration of LA and ARA were comparable between men and women and showed the same group differences as considering the whole study sample (Table 1).

**Fig. 3:**
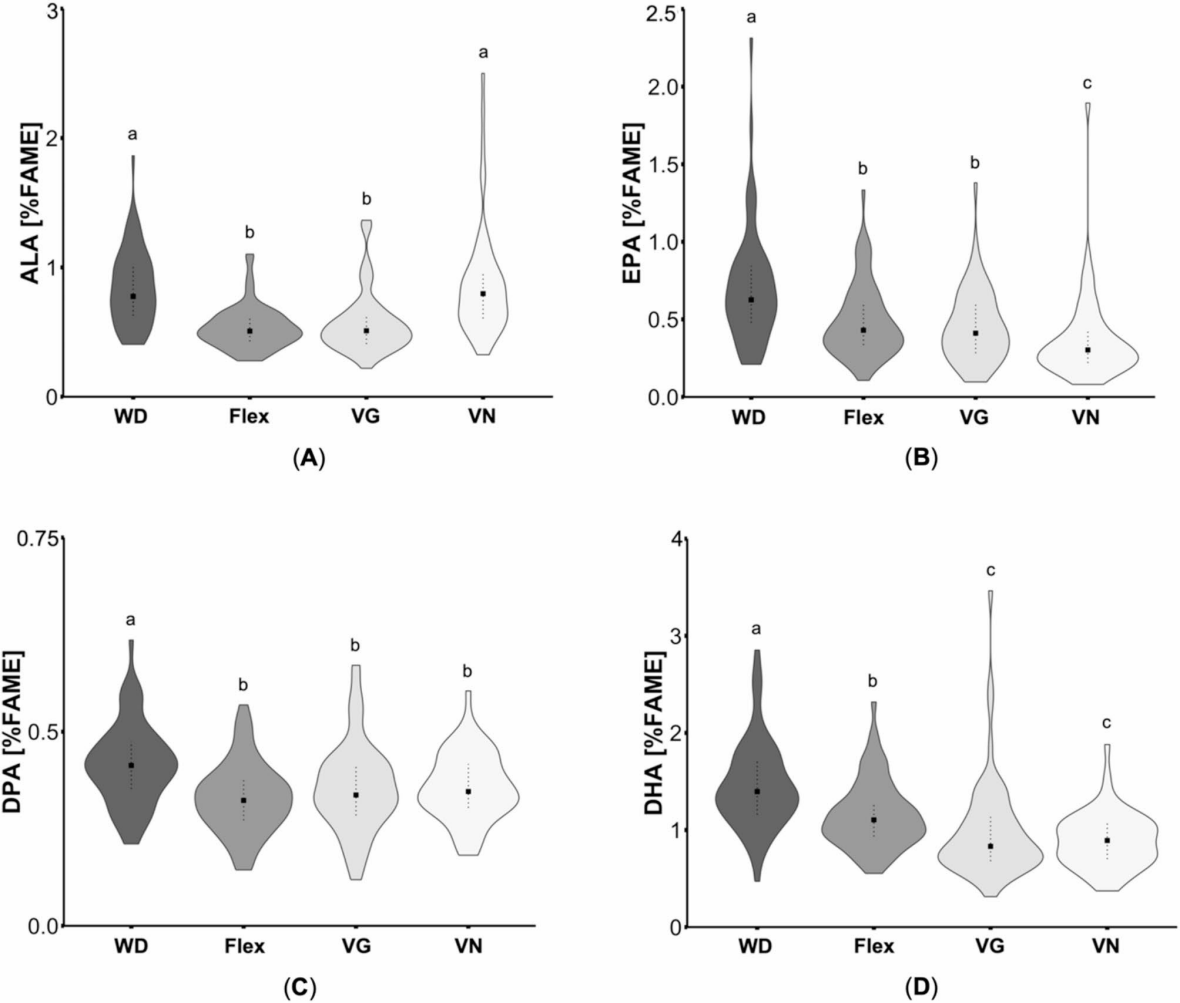
Comparison of selected plasma *n*-3 fatty acids of subjects with different dietary patterns. (**A**) α-Linolenic acid (ALA), (**B**) eicosapentaenoic acid (EPA), (**C**) docosapentaenoic acid (DPA) and (**D**) docosahexaenoic acid (DHA) concentrations in plasma lipids are presented as the percentage of the total area of total fatty acid methyl esters (%FAME). In total, 62 omnivores (Western diet; WD), 69 flexitarians (Flex), 64 vegetarians (VG) and 57 vegans (VN) were analyzed. The results are shown as violin plots with median (indicated as squares) and 25- and 75-quartile ranges (indicated as dotted lines). Diet groups that do not share indices (a, b,c) differ significantly (Kruskal-Wallis test followed by Benjamini-Hochberg correction (*p* < 0.05))

Regarding *n*-3 PUFA, flexitarians, vegetarians and vegans had lower proportions compared to omnivores (*p* ≤ 0.001). However, plasma ALA (C18:3*n*-*3*) of omnivores and vegans was slightly higher compared to flexitarians and vegetarians (*p* < 0.001, Fig. 3A). Concerning eicosatetraenoic acid (C20:4*n*-3), vegans showed lower proportions compared to omnivores (*p* < 0.001). For EPA (C20:5*n*-3), omnivores had the highest and vegans the lowest proportions which differ also from flexitarians and vegetarians (*p* ≤ 0.01, Fig. 3B). Furthermore, plasma DPA (C22:5*n*-3) was slightly higher in omnivores compared to all other groups (*p* < 0.001, Fig. 3C). DHA (C22:6*n*-3) also differed largely between the four groups with decreasing values in the following order: omnivores > flexitarians > vegetarians/vegans (*p* < 0.001; Table 1; Fig. 3D). In accordance, *n*-3 LC PUFA were significantly lower in vegetarians and vegans compared to omnivores (*p* < 0.001), while the proportion of those fatty acids was also reduced in vegans compared to flexitarians (*p* < 0.001). A comparative analysis of the data according to gender revealed comparable differences in ALA, EPA, and DHA between the groups as those identified in the overall study sample (Table 1).

The %*n*-6 HUFA was higher in vegetarians and vegans compared to omnivores and flexitarians (*p* ≤ 0.03), while omnivores also showed higher amounts than flexitarians (*p* = 0.02). In contrast, the group differences for %*n*-3 HUFA were reversed with decreasing amounts in the following order: omnivores > flexitarians > vegetarians/vegans (*p* ≤ 0.03; Table 1).

The sum of plasma trans fatty acids (TFA) was lower in vegans compared to all other groups (*p* < 0.001), whereby vegans had marginal higher amounts of trans-palmitoleic acid (C16:1t9) compared to omnivores (*p* < 0.05). However, vegans showed lower proportion of trans-octadecenoic acid (C18:1t9) in comparison to the other three groups (*p* < 0.001), with flexitarians and vegetarians also having lower rates than omnivores (*p* < 0.001). Furthermore, plasma levels of conjugated linoleic acids (CLA) were markedly lower in vegans compared to omnivores, flexitarians, and vegetarians (*p* ≤ 0.01), while flexitarians and vegetarians also had lower proportions than omnivores (*p* ≤ 0.01; Table 1).

Besides the analysis of single fatty acids, we also investigated different ratios which are related to inflammatory processes and consequently the cardiovascular risk. Plasma *n*-6 to *n*-3 ratio was significantly higher in flexitarians, vegetarians and vegans in comparison to omnivores, while vegans also showed higher proportions than flexitarians (*p* ≤ 0.001). Higher ratios in vegetarians and vegans were also evident for ARA to DHA (*p* < 0.001), while ARA to EPA was highest in vegans (*p* ≤ 0.01) and lowest in omnivores (*p <* 0.05) compared to all groups. LA to ALA ratio differed also between the groups with decreasing values in the following order: flexitarians/vegetarians > vegans > omnivores (*p <* 0.001). Moreover, vegan subjects had the lowest ratio of DPA to ALA and ARA to LA compared to all groups (both *p* ≤ 0.03), while flexitarians and vegetarians had higher proportions of DPA to ALA and lower proportions of ARA to LA compared to omnivores (both *p* ≤ 0.04; Table 1).

To validate the plasma fatty acids as short-term markers of fatty acid intake, correlation analyses were conducted between the calculated intake from the dietary record and the plasma fatty acids. The fatty acid intake can be found in detail in a prior publication [20]. Our data revealed a positive correlation between intake and plasma fatty acids for SFA (*r* = 0.395, *p* < 0.001), PUFA (*r* = 0.246, *p* < 0.001), *n*-6 PUFA (*r* = 0.165, *p* = 0.02), and *n*-3 PUFA (*r* = 0.198, *p* = 0.004). Oleic acid as well as total MUFA showed no significant correlation between the calculated intake and plasma levels. While EPA (*r* = 0.354, *p* < 0.001), ARA (*r* = 0.154, *p* = 0.03), and DHA (*r* = 0.358, *p* = 0.04) intake correlated with the respective plasma fatty acids levels, no correlation was found for DPA.

### Distribution of Fatty Acids in Erythrocytes

The sum of SFA in erythrocytes was slightly lower in flexitarians compared to omnivores (*p* = 0.04), with differences in individual SFA patterns also emerging between all dietary patterns. Myristic acid, pentadecanoic acid and heptadecanoic acid were lowest in vegans compared to all groups (*p* < 0.001). However, vegans had higher concentrations of stearic acid (C18:0) compared to flexitarians (*p* < 0.01), and the highest levels of arachidic acid (*p* < 0.001) compared to all three groups (Table 2).

Regarding MUFA, we found slightly higher total quantities in vegans compared to flexitarians and vegetarians (*p* < 0.01). However, palmitoleic acid levels were lowest in vegans compared to all groups (*p* < 0.001). Within MUFA, oleic acid was predominant in all dietary groups (15.7–16.4%FAME) and vegans had higher proportions compared to flexitarians and vegetarians (*p* = 0.02). Eicosenoic acid levels were highest in vegans compared to the other groups (*p* < 0.01). Proportions of eicosenoic acid were also higher in omnivores and vegetarians than in flexitarians (*p* < 0.01; Table 2).

**Fig. 4:**
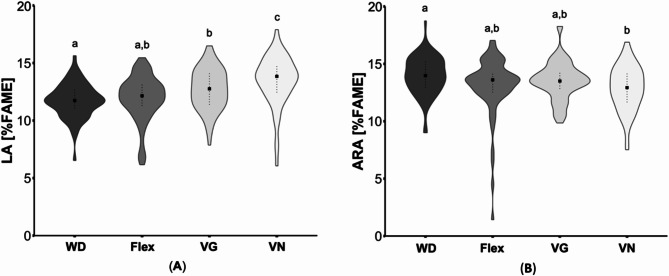
Comparison of selected *n*-6 fatty acids in erythrocyte lipids of subjects with different dietary patterns. (**A**) Linoleic acid (LA) and (**B**) arachidonic acid (ARA) concentrations in erythrocyte lipids were presented as the percentage of the total area of total fatty acid methyl esters (%FAME). In total, 62 omnivores (Western diet; WD), 69 flexitarians (Flex), 64 vegetarians (VG) and 57 vegans (VN) were analyzed. The results are shown as violin plots with median (indicated as squares) and 25- and 75-quartile ranges (indicated as dotted lines). Diet groups that do not share indices (a, b,c) differ significantly (Kruskal-Wallis test followed by Benjamini-Hochberg correction (*p* < 0.05))

Vegetarians and vegans had significantly higher *n*-6 PUFA levels compared to omnivores and flexitarians (*p* < 0.01). This group difference was particularly evident for LA (Fig. 4A), eicosadienoic acid, dihomoγlinolenic acid (C20:3*n*-6) and docosatetraenoic acid (all *p* ≤ 0.03), with LA and eicosadienoic acid showing the highest values in vegans, which also differ compared to vegetarians (both, *p* ≤ 0.02). Flexitarians and vegetarians had a higher proportion of γ-linolenic acid (C18:3*n*-6), than omnivores and vegans (*p* < 0.05). In addition, omnivores exhibited slightly higher values of ARA compared to vegans (*p* = 0.01; Fig. 4B). Notably, there was a group difference for *n*-6 docosapentaenoic acid (C22:5*n*-6), with vegetarians having the highest concentration compared to all groups (*p* < 0.001; Table 2).

Regarding sex specific differences, LA concentrations were only highest in male vegans (*p* < 0.01), whereas in both vegetarians and vegans, woman exhibited higher values compared to omnivores (both *p* ≤ 0.04). ARA concentrations were comparable between the women, whereas in men, similar to the entire collective, only vegans showed lower values (*p* < 0.001; Table 2).

**Fig. 5:**
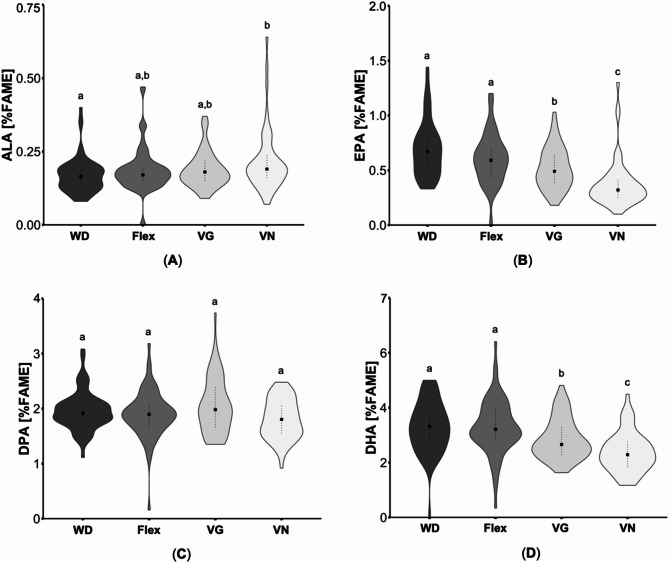
Comparison of selected *n*-3 fatty acids in erythrocyte lipids of subjects with different dietary patterns. (**A**) α-Linolenic acid (ALA), (**B**) eicosapentaenoic acid (EPA), (**C**) docosapentaenoic acid (DPA) and (**D**) docosahexaenoic acid (DHA) concentrations in erythrocytes were presented as the percentage of the total area of total fatty acid methyl esters (%FAME). In total, 62 omnivores (Western diet; WD), 69 flexitarians (Flex), 64 vegetarians (VG) and 57 vegans (VN) were analyzed. The results are shown as violin plots with median (indicated as squares) and 25- and 75-quartile ranges (indicated as dotted lines). Diet groups that do not share indices (a, b,c) differ significantly (Kruskal-Wallis test followed by Benjamini-Hochberg correction (*p* < 0.05))

The sum of *n*-3 PUFA was lowest in vegans compared to the other groups (*p* < 0.001). However, vegans had a slightly higher proportion of ALA than omnivores (*p* = 0.01; Fig. 5A). However, this difference was no longer observed when the collective was considered separately by gender (Table 2). For eicosatrienoic acid (C20:3*n*-3) omnivores as well as vegans had higher levels than vegetarians (*p* ≤ 0.03). In contrast, vegans had the lowest concentrations for eicosatetraenoic acid compared to the three groups (*p* < 0.001). DPA did not differ between the dietary patterns (Fig. 5C), but for both EPA and DHA, vegetarians had much lower values than omnivores (both *p* ≤ 0.001) and flexitarians (both *p* ≤ 0.03), with vegans having the lowest concentrations compared to all groups (both *p* < 0.001; Fig. 5B, D). EPA and DHA concentrations were only lower in male vegans compared to the omnivores groups (*p* < 0.001), while woman displayed the same differences as the whole collective (*p* < 0.001). Due to those differences in the *n*-3 PUFA pattern, vegetarians had a noticeably lower *n*-3 index than omnivores and flexitarians (*p* < 0.001), with vegans achieving the lowest values compared to all groups (*p* < 0.001). The LC *n*-3 PUFA sum was also significantly lower in vegans compared to the other groups (*p* < 0.001) and did further differ between vegetarians and omnivores (*p* < 0.001).

Overall, the sum of PUFA was equal between the groups, but the %*n*-6 HUFA was higher in vegetarians and vegans compared to omnivores and flexitarians (*p* ≤ 0.02), while the %*n*-3 HUFA was lower in both vegetarian groups (*p* ≤ 0.02; Table 2).

Regarding the sum of TFA, vegans had significantly lower values compared to omnivores, flexitarians, and vegetarians (*p* < 0.001). This is mainly due to the lower proportion of trans-octadecanoic acid (*p* < 0.001), whereas for trans-palmitoleic acid lower values were also observed in omnivores compared to flexitarians and vegetarians (*p* ≤ 0.02). Moreover, CLA were markedly lower in vegans compared to omnivores, flexitarians and vegetarians (*p* < 0.001; Table 2).

Regarding the different fatty acid ratios, *n*-6 to *n*-3 ratio was significantly higher in vegetarians and vegans in comparison to omnivores and flexitarians (*p <* 0.001), while vegans also showed higher values than vegetarians (*p <* 0.001). Higher ratios in vegetarians and vegans were also evident for the ratios ARA to EPA and ARA to DHA (all *p* < 0.05*)*. In contrast vegans had the lowest ratio of ARA to LA compared to all groups (*p <* 0.001), with flexitarians and vegetarians displaying a lower ratio compared to omnivores (*p* ≤ 0.04) (Table 2).

We correlated the individual erythrocyte fatty acids with the sum of SFA, MUFA as well as *n*-3 and *n*-6 PUFA to identify the fatty acids that best serve as biomarkers for the respective lipid classes. The correlation analysis revealed that the respective individual fatty acids that are represented the highest (%FAME) also have the strongest correlation to the respective sums (C16:0 and SFA, *r* = 0.560, *p* < 0.001; oleic acid and MUFA, *r* = 0.980, *p* < 0.001; ARA, C22:4*n*-6 and *n*-6 PUFA (*r* = 0.537, *r* = 0.699; both *p* < 0.001), EPA, DHA and *n*-3 PUFA (*r* = 0.810, *r* = 0.856, both *p* < 0.001).

### Comparison of inflammation-related Biomarkers

The cytokines IL-6, IL-8, IL-10 and TNF-α did not differ between omnivores, flexitarians, vegetarians and vegans (*p* > 0.05). Regarding hsCRP, vegans showed a tendency to have lower hsCRP levels compared to omnivores and flexitarians (*p* = 0.09). While leptin was reduced in vegans compared to omnivores (*p* < 0.05), adiponectin and the adiponectin/leptin ratio did not differ between the four dietary patterns (Table 3).

**Table 3:** Anti-inflammatory and inflammatory markers and adipokines according to diet group

	WD (23 m, 39 w)		Flex (14 m, 55 w)		VG (18 m, 46 w)		VN (17 m, 40 w)	
**IL-6 [pg/ml]**	0.74 / 2.34	a	0.81 / 1.60	a	1.15 / 2.24	a	0.91 / 1.81	a
**IL-8 [pg/ml]**	5.04 / 3.71	a	5.49 / 3.80	a	5.27 / 2.96	a	4.92 / 3.30	a
**IL-10 [pg/ml]**	2.07 / 3.05	a	2.49 / 2.09	a	2.28 /1.71	a	1.91 / 2.54	a
**TNF-**α **[pg/ml]**	2.65 / 7.08	a	2.93 / 2.92	a	3.20 / 3.43	a	2.78 / 3.02	a
**hsCRP [mg/l]**	0.60 / 1.55	a	0.70 / 0.93	a	0.55 / 0.93	a	0.40 / 0.30	a
**Adiponectin [ng/ml]**	80.09 / 97.77	a	71.25 / 49.93	a	88.36 / 66.23	a	71.77 / 55.82	a
**Leptin [ng/ml]**	7.39 / 13.81	a	6.06 / 9.58	a, b	5.12 / 6.05	a, b	3.73 / 8.03	b
**Adiponectin/Leptin**	9.23 / 27.95	a	13.41 / 24.82	a	14.00 / 29.84	a	13.33 / 49.86	a

Results are presented as median / IQR. Diet groups that do not share indices (a, b) differ significantly (Kruskal-Wallis test followed by Benjamini-Hochberg correction (*p* < 0.05)). Abbreviations: WD = omnivores, Flex = flexitarians, VG = vegetarians, VN = vegans, IL = interleukin, TNF-α = tumor necrosis factor α, hsCRP = high-sensitive C-reactive protein

**Fig. 6:**
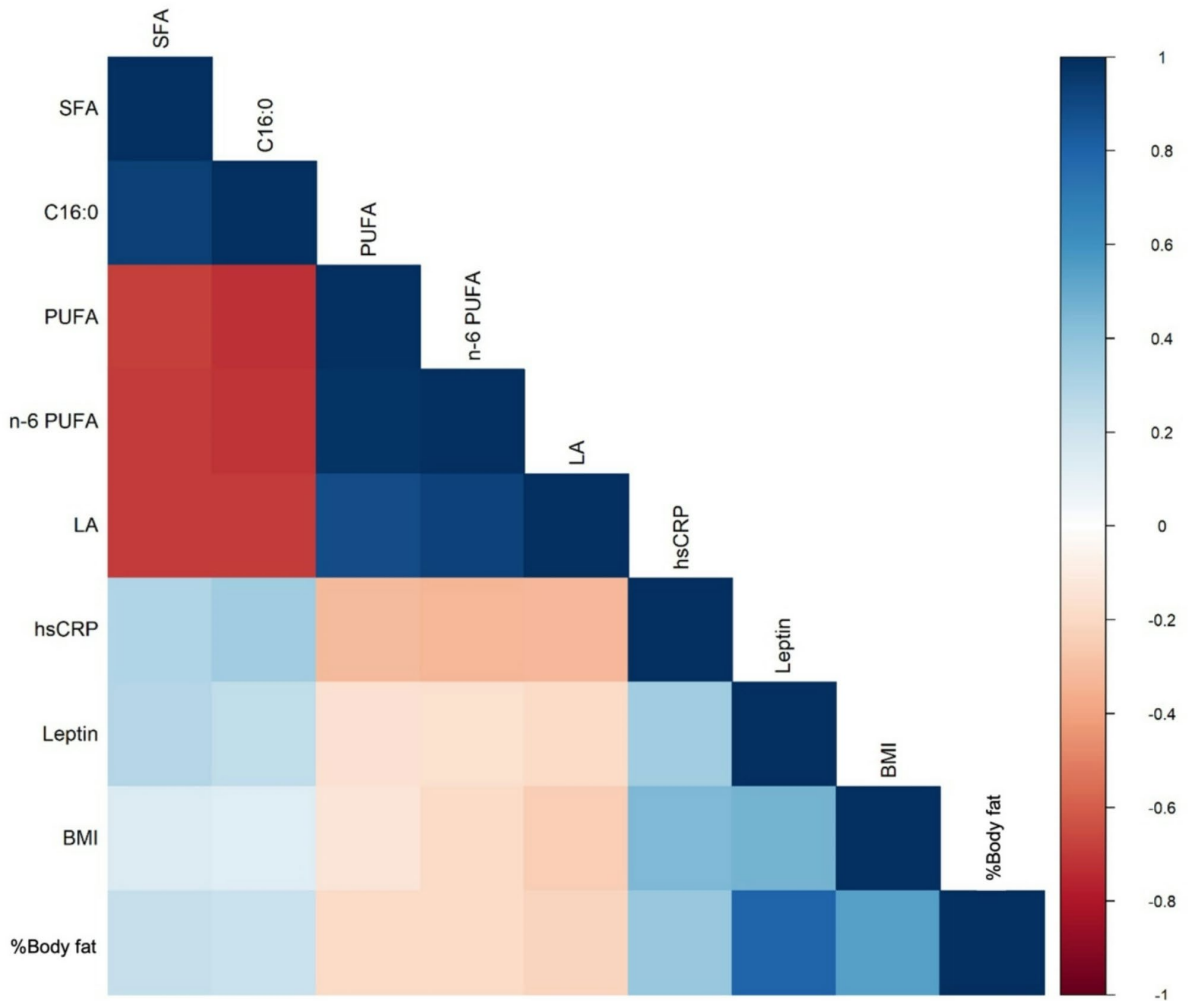
Correlation matrix of selected fatty acids in plasma, inflammatory markers, adipokines and parameters of body composition according to Spearman´s rank correlation. SFA = saturated fatty acids, PUFA = polyunsaturated fatty acids, *n* = omega, C16:0 = palmitic acid, LA = linoleic acid, hsCRP = high-sensitive C-reactive protein, BMI = body mass index

Furthermore, the correlation between inflammation-related biomarkers, selected fatty acids in both plasma and erythrocyte lipids, as well as body composition was analyzed. We found that only a few correlations were stronger pronounced for which a correlation matrix was generated (Fig. 6). In general, stronger correlations were observed in plasma compared to erythrocyte lipids, where hsCRP showed a positive correlation with total SFA (*r* = 0.296, *p* < 0.001), particularly with palmitic acid (*r* = 0.348, *p* < 0.001). Conversely, hsCRP was inversely correlated with plasma total PUFA (*r* = -0.320, *p* < 0.001) and *n*-6 PUFA (*r* = -0.333, *p* < 0.001), mainly driven by LA (*r* = -0.329, *p* < 0.001). Leptin was positively correlated with total SFA (*r* = 0.282, *p* < 0.001) and palmitic acid (*r* = 0.245, *p* < 0.001) in plasma. In addition, body mass index (BMI) and body fat percentage showed strong correlations with leptin (*r* = 0.463, *p* < 0.001; *r* = 0.798, *p* < 0.001) and hsCRP (*r* = 0.444, *p* < 0.001; *r* = 0.380, *p* < 0.001), further reinforcing the connection between body composition, adipokines and inflammation (Fig. 6).

Weaker correlations in plasma were shown for IL-6 and total MUFA, which exhibited a positive correlation (*r* = 0.172, *p* = 0.01), whereas IL-8 showed no significant correlation with any fatty acids in plasma lipids. TNF-α was negatively correlated with LA (*r* = -0.124, *p* < 0.05), EPA (*r* = -0.147, *p* = 0.02), and total PUFA (*r* = -0.124, *p* < 0.05), but positively correlated with total MUFA (*r* = 0.139, *p* = 0.03) and the ARA/EPA ratio (*r* = 0.151, *p* = 0.02). IL-10 was inversely correlated with EPA (*r* = -0.152, *p* = 0.02) and *n*-3 DPA (*r* = -0.153, *p* = 0.02), while the ARA/EPA ratio was positively correlated with IL-10 (*r* = 0.160, *p* = 0.014). A weaker positive correlation between hsCRP and total TFA (*r* = 0.208, *p* < 0.01) was found, along with inverse correlations for *n*-3 DPA (*r* = -0.184, *p* = 0.02), the *n*-6/*n*-3 ratio (*r* = -0.179, *p* = 0.02), and the ARA/DHA ratio (*r* = -0.168, *p* = 0.03). Leptin was also weakly positively correlated with TFA (*r* = 0.170, *p* < 0.01) and DHA (*r* = 0.158, *p* = 0.01), while negative correlations were observed with LA (*r* = -0.188, *p* < 0.01), DPA (*r* = -0.213, *p* < 0.001), total PUFA (*r* = -0.159, *p* < 0.01), *n*-6 PUFA (*r* = -0.163, *p* < 0.01), and the ARA/DHA ratio (*r* = -0.141, *p* = 0.03). No correlation was found between adiponectin and the selected fatty acids.

In erythrocytes, IL-6 showed a negative correlation with palmitic acid (*r* = -0.203, *p* < 0.01). IL-8 was positively correlated with *n*-3 DPA (*r* = 0.145, *p* < 0.01) and total *n*-3 PUFA (*r* = 0.135, *p* = 0.03), while the correlation with the *n*-6/*n*-3 ratio was inverse (*r* = -0.131, *p* = 0.04). In addition, TNF-α was positively associated with ARA (*r* = 0.176, *p* < 0.01), total *n*-6 PUFA (*r* = 0.150, *p* = 0.02), and the ARA/EPA ratio (*r* = 0.166, *p* < 0.01), while palmitic acid showed a negative correlation (*r* = -0.139, *p* = 0.028). IL-10 was positively correlated with total PUFA (*r* = 0.203, *p* < 0.01) and negatively with palmitic acid (*r* = -0.178, *p* < 0.01). hsCRP was positively correlated with palmitic acid (*r* = 0.226, *p* < 0.01) and total SFA (*r* = 0.170, *p* = 0.03), with a negative correlation with LA.

(*r* = -0.239, *p* < 0.01). Leptin showed positive correlations with palmitic acid (*r* = 0.173, *p* < 0.01), total SFA (*r* = 0.172, *p* < 0.01) and DHA (*r* = 0.130, *p* = 0.04), while inverse correlations were found with total MUFA (*r* = -0.130, *p* = 0.04), total *n*-6 PUFA (*r* = -0.127, *p* < 0.05), LA (*r* = -0.296, *p* < 0.001), *n*-3 DPA (*r* = -0.174, *p* < 0.01) and the ARA/DHA ratio (*r* = -0.136, *p* = 0.03). Adiponectin correlated positively with total PUFA (*r* = 0.174, *p* < 0.01), *n*-3 PUFA (*r* = 0.157, *p* = 0.02), ARA (*r* = 0.189, *p* < 0.01) and DHA (*r* = 0.150, *p* = 0.02).

Finally, weaker correlations were observed for BMI and body fat percentage, with inverse associations emerging with anti-inflammatory and inflammatory biomarkers such as IL-10 (*r* = -0.176, *p* < 0.01; *r* = -0.168, *p* = 0.01) and TNF-α (*r* = -0.127, *p* = 0.04; *r* = -0.186, *p* < 0.01). Adiponectin showed a weak positive correlation with BMI and lean mass (*r* = 0.218, *p* < 0.001; *r* = 0.128, *p* = 0.04).

## Discussion

There is a close link between the intake of dietary fats, and in particular their quality, and the risk of chronic diseases such as CVD [21]. Measuring circulating fatty acid composition as biological markers of dietary fat quality offers several advantages, as it is less susceptible to the typical errors associated with assessing intake, such as under/overreporting, changes in usual diet, incorrect portion sizes, variation in the fatty acid content of foods, and errors linked to the use of food composition databases, especially when considering individual fatty acids [22, 23]. However, due to the complexity of the fatty acid metabolism, it is not possible to derive specific amounts of fat intake based on the circulating fatty acids, but rather to provide general indications of the status. Moreover, the fatty acids are described as %FAME, which is the most common measurement, but this could potentially obscure differences in absolute concentrations. The NuEva collective is well suited to investigate the relationship between dietary fat intake as a function of the type of diet and circulating fatty acids. To provide a comprehensive overview of fatty acid profiles, we both investigated plasma and erythrocyte lipids to examine dietary adaptations.

Plasma SFA levels are influenced by current dietary intake. In agreement with previous reports, we confirm a moderate positive correlation between SFA in plasma lipids and dietary SFA consumption [24, 25]. Due to abstaining from meat and dairy products in vegan diets, plasma SFA, especially palmitic acid, which accounts for the largest amount of plasma SFA, is lower in vegans. This is further validated by lower levels of myristic acid and odd-numbered fatty acids (C15:0; C17:0) in the plasma of vegans. Since the odd-chain fatty acids are primarily considered as biomarkers for milk and ruminant fat intake [26, 27], our data may reflect compliance with the vegan diet. In recent years, it has been suggested that pentadecanoic and heptadecanoic acids can be endogenously synthesized from propionate, a product of soluble dietary fibre degradation [28], which may explain the lower but detectable levels of these odd-chain fatty acids in vegans. While the intake of SFA differs significantly between the four diets under investigation, the absence of differences in the sum of erythrocyte SFA levels between dietary patterns suggests that SFA are incorporated into membranes in a regulated manner, likely depending more on endogenous synthesis than on dietary intake [29].

No correlation was found between MUFA intake, particularly oleic acid as the most prominent MUFA, and plasma fatty acids. Oleic acid is found in large amounts in olive oil, which is commonly associated with the Mediterranean diet [30]. Since oleic acid can be synthesized endogenously it has not been considered to be a good marker of dietary oleic acid [31]. This may also explain the comparable plasma values across the four investigated groups. In contrast, earlier intervention studies involving modified MUFA intake have demonstrated that an elevated intake results in an augmentation of oleic acid in both plasma cholesterol and phospholipids [32, 33]. There is also evidence that regular consumption of olive oil as part of a Mediterranean diet leads to higher oleic acid levels in the erythrocyte membrane [34]. Therefore, the slightly higher oleic acid concentrations in erythrocyte lipids of the vegans could possibly be attributed to a more frequent consumption of foods rich in oleic acid such as olive oil or rapeseed oil.

The total amount of PUFA in plasma was highest in vegans compared to all groups, reflecting their higher dietary intake in plant-based diet [9–11]. In contrast, total PUFA in erythrocyte lipids did not differ between the groups, which may be because their incorporation into the membrane is more tightly regulated to maintain the physical functions of the cell membrane [35]. However, the differences in *n*-6 and *n*-3 PUFA levels between the four dietary patterns were comparable for both plasma and erythrocyte lipids. In all four diets, LA accounts for the largest percentage of *n*-6 fatty acids, ranging from 29.2 to 35.6 in plasma lipids and from 11.7 to 13.8 in erythrocyte lipids. Elevated plasma and erythrocyte *n*-6 PUFA levels in vegans are therefore mainly due to an higher intake of LA, a fatty acid abundant in plant foods like nuts (e.g., brazil nuts, walnuts, pine nuts), seeds (e.g., hemp), certain vegetables, and oils (e.g., soybean oil, sunflower oil) [36]. ARA is the main product of *n*-6 PUFA biosynthesis and is mainly found in animal foods such as meat and egg yolk [37], which explains the higher plasma levels in omnivores. The lack of ARA in vegan diets is evident in both plasma and erythrocytes as this group had the lowest ARA levels.

ALA is predominantly found in plant foods such as flaxseed, chia seeds, hemp seeds, walnuts, and their oils. Therefore vegans and vegetarians may have a higher intake [38]. However, our study did not find significant differences in plasma ALA concentrations, but slightly higher levels in the erythrocyte lipids of vegans compared to omnivores. This might indicate an adaptation to a plant-based diet, potentially as a compensatory mechanism to maintain membrane fluidity in the absence of LC *n*-3 fatty acids or a higher regular intake of ALA, leading to more efficient incorporation into phospholipids instead of $\:\beta\:$-oxidation, which is the main metabolic pathway of ALA [39]. In addition, the LA/ALA ratio in plasma differed significantly between the groups, whereas the ratio was comparable in erythrocytes suggesting that the incorporation of both fatty acids in cell membrane is strongly regulated. Previous studies reported an inverse correlation of the LA/ALA ratio with EPA and DHA, especially in vegans [40]. This underlines that the endogenous conversion of ALA to LC *n*-3 PUFA seems to be limited in a plant-based diet, which is usually rich in *n*-6 PUFA, due to the competition of *n*-3 and *n*-6 PUFA for the respective rate-limiting desaturases [40]. The same group differences occurred when considering women separately. In the literature it is stated that woman exhibit a higher conversion of ALA to EPA and DHA due to lower β-oxidation rate of ALA and increased activity of desaturases and elongases, influenced by higher estrogen levels [39, 41]. However, we did not observe major sex-specific differences in ALA, EPA, or DHA between the groups. This is likely due to the uneven gender distribution in our study, where most participants were female (70%), reducing the potential for meaningful sex-based comparisons.

Overall, omnivores had higher plasma *n*-3 PUFA levels, particularly EPA and DHA, corresponding to their intake of fish, which is absent in vegetarian and vegan diets. These group differences are also reflected in erythrocyte lipids, although the effects are not as pronounced as in plasma, suggesting that endogenous conversion from ALA to EPA and DHA occurs even in plant-based diets, despite a high LA intake.

This is also evident in the DPA levels in erythrocytes, which were comparable across all groups. DPA serves as an intermediary in this conversion process and remains efficiently integrated into erythrocyte membranes, possibly contributing to membrane stability and functioning over time [42]. The lack of correlation between DPA intake and its concentration in plasma lipids suggests that endogenous synthesis from ALA or EPA may play a more significant role than dietary intake. Moreover, it cannot be ruled out that the values recorded in the databases for DPA do not accurately reflect the actual content in foods.

Our results showed that vegans had lower levels of total TFA in both erythrocytes and plasma than the other groups, indicating a lower dietary intake. TFA can be classified as natural (of ruminal origin) or industrially produced, with the latter resulting from the partial hydrogenation or deodorization of MUFA [43]. Besides the intake from ruminant products, ultra-processed foods account for the majority of dietary TFA [43]. However, the ingredients and manufacturing processes of ultra-processed meat and dairy substitutes vary greatly [44]. Our data confirms the avoidance of ruminant-based foods in vegans and further indicate for a low consumption of ultra-processed meat and dairy substitutes.

In accordance, we found that CLA in erythrocytes and plasma of vegans was less than half of the concentration of omnivores. The intake of CLA is primarily derived from dairy and ruminant meat products [45], thus the lower levels in plasma likely reflect the short-term differences in the consumption of dairy products between omnivores and vegans. The long-term exclusion of dairy products by vegans was reflected by the differences in CLA concentrations in erythrocyte lipids.

In summary, our data confirms that analysis of circulating fatty acids in plasma lipids is well-suited for assessing short-term fatty acid intake over the last few days. This analysis also reflects changes in SFA intake, which could not be depicted by analysis of the fatty acid distribution in erythrocyte lipids. This indicates that the SFA concentration in the phospholipids or membranes is endogenously regulated. Except for SFA intake, the main characteristics in fatty acid intake in dependence of the relation between plant- and animal-based food were reflected by changes in both plasma and erythrocyte lipids. Thus, our data suggests that erythrocytes can provide insights into more regular intake as well as the endogenous conversion and integration of fatty acids into cell membranes, especially for MUFA, *n*-6, and *n*-3 PUFA, including *n*-3 DPA. In general, erythrocyte fatty acids reflect mid-term dietary intake due to a gradual turnover of erythrocytes of around 120 days, but their composition is also subject to dynamic regulation and can respond to dietary changes within shorter timeframes [46, 47], which must be considered. Therefore, the combination of both biomarkers reflecting different timeframes provides a comprehensive overview of dietary fat intake in relation to dietary patterns.

Overall, the differences we found for SFA, MUFA, *n*-6, and especially LC *n*-3 PUFA between the dietary patterns are consistent with previous studies that examined fatty acid composition in omnivores, vegetarians, and vegans in plasma or serum and erythrocytes [40, 48–64]. To further contextualize our findings, we have compiled a comparative table summarizing key *n*-6 and *n*-3 PUFA (LA, ARA, ALA, EPA, DHA) from our study alongside similar studies in the literature (TableS1). This comparison highlights that characteristic fatty acid profiles are linked to dietary habits, despite methodological differences across studies. Thus, our data align well with the existing literature, reaffirming that fatty acid profiles differ especially between omnivores and vegans.

In the next step, we aim to investigate the extent to which the described difference in the fatty acid profiles influence the inflammatory process in the NuEva collective. An imbalance of *n*-6/*n*-3 is associated with potential health implications such as CVD and proinflammatory conditions [65]. Although several scientists emphasize the relevance of *n*-6/*n*-3 balance, there are no cutoff markers in erythrocytes and plasma that would indicate a deficient *n*-3 status [64]. In the NuEva collective, the highest *n*-6/*n*-3 ratio was observed among vegans.

An increased abundance of EPA and DHA in cell membrane phospholipids is associated with anti-inflammatory effects by reducing the production of pro-inflammatory eicosanoids from ARA and synthesis of specialized pro-resolving mediators, while also influencing other processes involved in inflammation, such as lipid raft formation, gene transcription regulation and cytokine (chemokine and adhesion molecules) production [66, 67]. As EPA competes simultaneously with ARA for the key enzymes cyclooxygenase and lipoxygenase to form less proinflammatory mediators, the use of the ARA/EPA ratio is discussed as a marker of chronic inflammation [68]. In the NuEva collective, vegans exhibited the highest ARA/EPA ratio in plasma lipids, followed by flexitarians and vegetarians, with omnivores showing the lowest ratio. In erythrocytes, no significant differences were found between flexitarians and omnivores, while vegetarians and vegans showed higher ARA/EPA ratios. These findings are in line with previous studies [57, 64]. The ARA/DHA ratio is much less described in the literature and, to the best of our knowledge, has not yet been compared between different dietary patterns, but our data suggest that predominantly plant-based diets are characterized by higher ARA/DHA ratios in comparison to omnivores. Overall, these results highlight potential negative effects of low *n*-3 PUFA status in plant-based diets in the context of inflammation. To investigate physiological consequences, we also examined selected inflammation markers, which act as intermediate risk factors for the development of chronic diseases. Despite the lower *n*-3 PUFA status of vegans and vegetarians, no group differences emerged for the investigated inflammatory and anti-inflammatory biomarkers (hsCRP, TNF-ɑ, IL-6, IL-8, IL-10). In the Risk and Benefits of a Vegan Diet study, no significant differences in the inflammatory biomarkers, including hsCRP, were found between vegans and omnivores [69]. In accordance with our data, the authors showed trends for the dependence between inflammatory biomarkers and circulating SFA and PUFA level. While plasma SFA inversely correlated with the anti-inflammatory biomarker adiponectin, higher levels of PUFA were associated with lower levels of hsCRP [69]. Here, this was also shown for total PUFA, especially LA, and hsCRP, whereas the SFA palmitic acid negatively correlated with hsCRP. Further, we found a weak positive association of total PUFA with the anti-inflammatory biomarker IL-10 but also the proinflammatory agent TNF-ɑ. The latter is mainly due to *n*-6 PUFA, especially ARA, which also correlated with TNF-ɑ. However, the LC *n*-3 PUFA did not correlate with any of the inflammatory biomarkers investigated.

The balance between different fatty acids, particularly the ratio of *n*-6 to *n*-3 PUFA and the total concentration of PUFA, appears to be a determinant of the inflammatory profile. Despite a lower *n*-3 PUFA status among vegans and vegetarians, these diets do not result in a deterioration of the inflammatory profile in comparison to omnivores. This is in line with the fact that plant-based diets are discussed to prevent or counteract inflammatory state underlying numerous chronic diseases [70, 71]. Previous meta-analyses have also indicated that plant-based diets are associated with lower hsCRP levels compared to meat-based diets, while no significant effects were observed for the markers IL-6, TNF-α, and adiponectin [72–74]. Here we also did not find group differences between those three markers, but vegans had almost 50% lower leptin concentrations compared to omnivores. Previous studies also showed reduced leptin concentrations in vegetarians and vegans, which is most likely due to the differences in body composition. In earlier reports, leptin was found to have a positive correlation and adiponectin a negative correlation with BMI and body fat content [75, 76]. In the present study, however, the correlation could only be reproduced for leptin. Adiponectin was found to correlate with lean mass. The adipokines adiponectin and leptin have opposing effects on inflammation. While leptin stimulates the production of cytokines like IL-6 and TNF-α, adiponectin suppresses the expression and release of pro-inflammatory immune mediators exhibiting anti-inflammatory properties [77]. Moreover, the adiponectin/leptin ratio has been identified as an indicator of adipose tissue function. Previous studies have indicated a negative correlation between this ratio and markers of low-grade chronic inflammation, such as hsCRP [78, 79]. However, this was not observed in the NuEva collective. We also showed a strong association between BMI, body fat percentage and hsCRP. Given the strong correlation between obesity and low-grade inflammation [80], it is plausible that the lower BMI and body fat percentage observed in vegans and vegetarians exerts an influence on the inflammatory state, even if this is not reflected in every marker. This highlights the complexity of the relationship between dietary patterns and inflammation. Regarding fatty acids, the low SFA [81] and high PUFA [82] intake in a vegetarian or vegan diet is generally beneficial in the context of inflammation. In addition, a plant-based diet usually consists of foods rich in antioxidant micronutrients [83, 84], which are known for their anti-inflammatory properties [85]. Moreover, other factors, such as the duration of adherence to a plant-based diet (at least two years), may also exert a significant influence on inflammatory markers such as hsCRP [69, 72]. Overall, the NuEva study consisted of healthy subjects, so it was expected that few differences in inflammatory markers would be observed, given that such markers are physiologically tightly regulated [86].

Nevertheless, the anti-inflammatory and inflammation-resolving properties of EPA and DHA are important for both the prevention and treatment of diseases with an inflammatory component. This has been extensively researched in rheumatoid arthritis, where a low *n*-3 PUFA intake has been identified as a contributing factor in the etiology [87]. Moreover, substantial evidence indicates that EPA and DHA can mitigate the infiltration of inflammatory cells and the production of inflammatory mediators, thereby reducing pain and other symptoms [88–90]. It has been shown that a regular intake of approx. 3 g EPA + DHA per day reduces the intensity of the disease and therefore supports the therapy [89, 91]. It is therefore recommended to ensure an adequate supply of *n*-3 PUFA, to reduce development and progress of inflammatory diseases.

## Strengths and Limitations.

Overall, this study provides a comprehensive fatty acid profile of plasma and erythrocytes in healthy individuals following different dietary patterns, offering valuable insights into both nutrient status and the use of blood fatty acids as biomarkers of dietary-induced differences of fat intake. The inclusion of four distinct dietary groups reflects current dietary trends, particularly the growing interest in plant-based nutrition. By assessing inflammatory markers alongside fatty acid biomarkers, the study provides insight into potential metabolic differences or adaptations associated with different dietary habits.

However, certain limitations must be considered. The study population has an uneven sex distribution, with more women than men, which may limit generalizability. Additionally, the wide age range of participants prevents meaningful stratification by age groups. A further limitation is the reliance on a self-reported dietary protocol, which is subject to reporting biases, including the tendency to over- and underreporting. This is of particular interest regarding the consumption of fat, since it is challenging to accurately estimate the quantity and type of fat utilized in the preparation of meals when eating out. Moreover, challenges in the estimation of dietary fat intake exists, such as discrepancies between fatty acid profiles calculated by software and the actual fatty acid composition of consumed foods. Finally, it should be noted that the NuEva subjects were healthy individuals who did not have any inflammatory diseases, which limits the ability to assess the impact of fatty acid intake on the inflammatory response.

## Conclusions

Our results underline the importance of considering both plasma and erythrocyte fatty acids as complementary biomarkers for assessing fatty acid intake and metabolism. Plasma fatty acids are more sensitive to recent dietary intake, while erythrocyte fatty acids provide insights into longer-term dietary patterns and the influence on membrane lipid composition. The observed differences in fatty acid profiles between omnivores and vegans are also in line with existing literature, confirming higher levels of *n*-6/*n*-3 due to high LA and lower EPA and DHA levels. Although there were no significant differences in inflammatory markers, the higher ratios of ARA/EPA and ARA/DHA observed in vegans and vegetarians may indicate a lower ability to produce eicosanoids and resolvins from EPA and DHA, which have anti-inflammatory and pro-resolving effects.

## Electronic supplementary material

Below is the link to the electronic supplementary material.

Supplementary Material 1

## Data Availability

No datasets were generated or analysed during the current study.

## Abbreviations

ALA: α-linolenic acid
ARA: arachidonic acid
BMI: body mass index
CLA: conjugated linoleic acids
CVD: cardiovascular disease
DHA: docosahexaenoic acid
DPA: docosapentaenoic acid
EPA: eicosapentaenoic acid
FAME: fatty acid methyl ester
hsCRP: high-sensitive C-reactive protein
HUFA: highly unsaturated fatty acids
IL: interleukin
IQR: interquartile range
LA: linoleic acid
LC: long-chain
MUFA: monounsaturated fatty acids
n: omega
NuEva: Nutritional Evaluation
PUFA: polyunsaturated fatty acids
SFA: saturated fatty acids
TNF-α: tumor necrosis factorα
TFA: trans fatty acids
