# Supplementary material for: Comparative analysis of fatty acid profiles across omnivorous, flexitarians, vegetarians, and vegans: insights from the NuEva study
Source: Lipids Health Dis. 2025 Apr 9;24:133. doi: 10.1186/s12944-025-02517-6 (PMC11983864; doi:10.1186/s12944-025-02517-6)
Supplement: Supplementary file 1 — Supplementary Material 1 [file 12944_2025_2517_MOESM1_ESM.docx]

**Table S1:** Overview of the fatty acid profile across different dietary patterns in different biomarkers [1-18]

| **Study** | **Biomarker** | **Subjects** | **n** | **LA** | **ARA** | **ALA** | **EPA** | **DHA** |
| --- | --- | --- | --- | --- | --- | --- | --- | --- |
| **Plasma, serum or whole blood** | | | | | | | | |
| Sanders et al. 1978^1^ | Plasma Phosphatidylcholine  (mg/g) | **Australia**  Omnivor  Vegan | 22  22 | 260 ± 7.1^a^  333 ± 11.5^b^ | 91 ± 4.1^a^  106 ± 5.5^b^ | ND | 13 ± 0.9^a^  2 ± 0.2^b^ | 40 ± 2.5^a^  14 ± 1.7^b^ |
| Dickerson et al. 1979^2^ | Plasma choline phosphoglycerides (mg/g) | **Germany**  Omnivor  Vegan | 12  12 | 260^a^  333^b^ | 91^a^  106^b^ | ND | 13^a^  2^b^ | 40^a^  14^b^ |
| Melchert et al. 1987^1^ | Total serum lipids  (% total fatty acids) | **Germany**  Men  Omnivor  Vegetarian  Women  Omnivor  Vegetarian | 38  37  62  70 | 30.07 ± 4.99^a^  36.92 ± 6.60^b^  30.98 ± 4.93^a^  36.29 ± 5.26^b^ | 6.35± 1.28^a^  5.65 ± 1.43^a^  6.35 ± 1.36^a^  6.08 ± 1.48^a^ | 1.05 ± 0.25^a^  1.62 ± 1.89^b^  1.10 ± 0.35^a^  1.35 ± 0.81^b^ | ND | 2.15 ± 0.53^a^  1.20 ± 0.46^b^  2.36 ± 0.59^a^  1.42 ± 0.47^b^ |
| Lee et al.  2000^1^ | Total serum lipids  (% of total fatty acids) | **China**  Omnivore  Vegetarian | 133  60 | 29.5 + 7.6^a^  38.2 + 4.1^b^ | 6.3 + 2.2^a^  5.3 + 1.6^b^ | 0.8 + 0.6^a^  1.7 + 2.0^b^ | 1.3 + 1.3^a^  0.2 + 0.5^b^ | 3.4 + 2.2^a^  1.7 + 2.5^b^ |
| Fokkema et al. 2000^1^ | Plasma triglycerides (mol%) | **Netherlands**  Omnivor  Vegan | 15  12 | 18.55 + 2.83^a^  26.85 + 4.17^b^ | 1.47 +0.62^a^  1.41 +0.29^a^ | 1.20+0.38^a^  1.47+0.70^a^ | 0.25 + 0.15^a^  0.14 + 0.10^a^ | 0.72 + 0.44^a^  0.25 + 0.17^b^ |
| Rosell et al. 2005^3^ | Total plasma lipids  (% of total fatty acids) | **UK**  Omnivor  Vegetarian  Vegan | 232  231  196 | 30.42 (29.17, 31.67)^a^  33.59 (32.55, 34.62)^b^  37.12 (36.04, 38.21)^c^ | 5.77 (5.50, 6.04)^a^  5.63 (5.41, 5.86)^a^  5.82 (5.58, 6.05)^a^ | 1.30 (1.20, 1.41)^a^  1.39 (1.30, 1.48)^a^  1.41 (1.32, 1.50)^a^ | 0.72 (0.65, 0.80)^a^  0.52 (0.48, 0.57)^b^  0.34 (0.31, 0.37)^c^ | 1.69 (1.59, 1.79)^a^  1.16 (1.07, 1.24)^b^  0.70 (0.61, 0.79)^c^ |
| Mann et al. 2006^1^ | Plasma phospholipids (mg/100g) | **Australia**  Omnivor  Flexitarian  Vegetarian  Vegan | 18  60  43  18 | 20.6 ± 2.6^a^  22.4 ± 2.8^b^  26.0 ± 3.1^c^  26.1 ± 2.5^c^ | 10.6 ± 1.6^a^  10.5 ± 1.7^a^  9.5 ± 1.9^b^  10.6 ± 1.5^a^ | 0.2 ± 0.1^a^  0.2 ± 1.0^a^  0.3 ± 0.1^b^  0.3 ± 0.1^b^ | 1.1 ± 0.5^a^  1.0 ± 0.3^a^  0.7 ± 0.3^b^  0.6 ± 0.3^b^ | 3.4 ± 1.0^a^  3.3 ± 0.8^a^  2.2 ± 0.7^b^  2.0 ± 0.4^b^ |
| Welch et al. 2010^1^ | Plasma phospholipids (µmol/L) | **UK**  Men  Omnivor  Omnivor, no fish  Vegetarian  Vegan  Women  Omnivor  Omnivor, no fish  Vegetarian  Vegan | 2646  2257  359  25  5  2256  1891  309  51  5 | 1164.1 ± 329.5  1207.9 ± 333.3  1238.2 ± 421.6  1337.7 ± 414.1  *p* < 0.001*  1236.9 ± 328.4  1271.2 ± 373.9  1325.9 ± 278.6  1406 ± 162.1  *p* < 0.001* | ND | 10.9 ± 5.7  11.8 ± 7.0  13.6 ± 10.1  15.8 ± 9.7  *p* < 0.001*  12.4 ± 6.1  13.1 ± 7.3  12.3 ± 4.8  13.7 ± 8.1  n.s | 57.5 ± 43.2  47.4 ± 30.3  55.9 ± 45.3  65.1 ± 45.5  *p* = 0.001*  64.7 ± 43.4  57.1 ± 38.4  55.1 ± 52.5  50.0 ± 29.4  *p* = 0.001* | 239.7 ± 106.2  215.6 ± 96.4  222.2 ± 138.4  195.0 ± 58.8  *p* < 0.001*  271.2 ± 113.1  241.3 ± 109.6  223.5 ± 137.8  286.4 ± 211.7  *p* < 0.001* |
| Elorinne et al. 2016^1^ | Total serum lipids  (% of total fatty acids) | **Finland**  Omnivor  Vegan | 18  22 | 31.74 ± 1.21^a^  36.77 ± 3.78^b^ | 6.87 ± 1.14^a^  6.27 ± 1.40^a^ | 0.73 ± 0.35^a^  1.28 ± 0.58^a^ | 2.33 ± 1.60^a^  0.63 ± 0.28^b^ | 2.25 ± 0.80^a^  0.85 ± 0.30^b^ |
| Pinto et al.  2019^3^ | Plasma  (% of total fatty acids) | **UK**  Omnivor  Vegan | 24  25 | 27.1 (26.0, 28.2)^a^  33.1 (31.9, 34.4)^b^ | 6.68 (8.12, 7.25)^a^  6.55 (5.94, 7.16)^a^ | 0.53 (0.48, 0.59)^a^  0.71 (0.59, 0.85)^b^ | 1.03 (0.79, 1.34)^a^  0.47 ( 0.40, 0.55)^b^ | 2.23 (1.94, 2.57)^a^  0.91 (0.80, 1.05)^b^ |
| Salvador et al. 2019^4^ | Total serum lipids  (% of total fatty acids) | **Spain**  Vegetarian  Vegan | 49  55 | 34.60 / 5.12^a^  34.09 / 4.93^a^ | 8.19 / 1.99^a^  8.08 / 2.59^a^ | 0.46 / 0.34^a^  0.49 / 0.32^a^ | 0.30 / 0.19^a^  0.24 / 0.17^a^ | 1.85 / 0.79^a^  1.40 / 0.93^b^ |
| Chamorro et al. 2020^1^ | Total plasma lipids (Mol%) | **Chile**  Omnivor  Vegan | 33  34 | 33.7 ± 4.5^a^  34.5 ± 5.10^a^ | 6.27 ± 1.60^a^  4.82 ± 1.52^b^ | 0.69 ± 0.1^a^  0.95 ± 0.29^b^ | 0.50 ± 0.2^a^  0.28 ± 0.13^b^ | 1.56 ± 0.40^a^  0.64 ± 0.26^b^ |
| Craddock et al.  2021^1,5^ | Whole blood fatty acids (% of total fatty acids) | **Autralia**  Omnivor Athletes  Vegan Athletes | 8  12 | 20.64(18.77–22.52)^a^  24.82(23.40–26.24)^b^ | 9.44(8.21–10.68)^a^  8.63(7.94–9.32)^a^ | 0.58(0.48–0.63)^a^  0.68(0.61–0.90)^b^ | 0.87(0.61–1.14)^a^  0.56(0.45–0.68)^b^ | 2.70(2.08–3.33)^a^  1.90(1.47–2.33)^b^ |
| Menzel et al. 2022^3^ | Plasma phospholipids (% of total fatty acids) | **Germany**  Omnivor  Vegan | 36  36 | 21.9 (21.3–22.6)^a^  25.7 (24.9–26.5)^b^ | 8.78 (8.20–9.40)^a^  8.23 (7.68–8.81)^a^ | 0.25 (0.22–0.27)^a^  0.27 (0.24–0.29)^a^ | 0.96 (0.83–1.11)^a^  0.49 (0.42–0.56)^b^ | 2.90 (2.63–3.19)^a^  1.50 (1.36–1.65)^b^ |
| Chamorro et al.  2023^1^ | Plasma phospholipids (Mol%) | **Chile**  Omnivor  Pescetarian  Vegan | 35  36  35 | 29.9 ± 4.1^a^  25.9 ± 3.3^a^  34.2 ± 4.9^a^ | 6.49 ± 1.8^a^  6.27 ± 0.8^a^  4.19 ± 0.3^b^ | 0.62 ± 0.1^a^  2.05 ± 0.3^b^  4.17 ± 0.4^c^ | 0.79 ± 0.1^a^  2.52 ± 0.4^b^  0.39 ± 0.04^c^ | 1.53 ± 0.3^a^  5.27 ± 0.4^b^  0.78 ± 0.1^c^ |
| Gogga et al. 2024^1^ | Total serum lipids  (% of total fatty acids) | **Poland**  Omnivor  Pescetarian  Vegetarian  Vegan | 29-30  9-13  27-28  29-30 | 28.05 ± 3.99^a^  30.73 ± 1.41^b^  27.90 ± 0.39^a^  31.12 ± 3.93^b^ | 3.66 ± 0.90^a^  3.04 ± 1.09^a,b^  2.62 ± 0.98^b^  2.54 ± 0.99^b^ | 0.17 ± 0.08^a^  0.14 ± 0.05^a,b^  0.11 ± 0.06^b^  0.15 ± 0.06^a,b^ | 0.34 ± 0.12^a^  0.30 ± 0.10^a^  0.23 ± 0.11^b^  0.21 ± 0.10^b^ | 0.63 ± 0.23^a^  0.74 ± 0.36^a^  0.48 ± 0.27^b^  0.22 ± 0.17^c^ |
| Klein et al.  2025^4^ | Total plasma lipids  (% of total fatty acids) | **Germany**  Omnivor  Flexitarian  Vegetarian  Vegan | 62  69  64  57 | 29.20 / 4.92^a^  30.66 / 5.67^b^  31.52 / 4.49^b^  35.57 / 6.04^b^ | 6.57 / 1.82^a^  5.43 / 1.80^b^  5.34 / 1.57^b^  5.20 / 1.79^b^ | 0.78 / 0.41^a^  0.51 / 0.20^b^  0.50 / 0.22^b^  0.80 / 0.38^a^ | 0.63 / 0.38^a^  0.43 / 0.28^b^  0.42 / 0.33^b^  0.30 / 0.23^b^ | 1.40 / 0.57^a^  1.11 / 0.39^b^  0.84 / 0.48^c^  0.89 / 0.38^c^ |
| **Erythrocyte** | | | | | | | | |
| Sanders et al.  1978^1^ | Erythrocyte lipids (mg/g) | **Australia**  Omnivor  Vegan | 22  22 | 87 ± 3.5^a^  113 ± 4.2^b^ | 125 ± 2.4^a^  126 ± 4.2^b^ | ND | 8 ± 0.7^a^  1 ± 0.2^b^ | 58 ± 3.8^a^  19 ± 2.3^b^ |
| Dickerson et al.  1979^2^ | Erythrocyte lipids (mg/g) | **Germany**  Omnivor  Vegan | 18  18 | 87^a^  113^b^ | 125^a^  126^a^ | ND | 8^a^  1^b^ | 58^a^  19^b^ |
| Agren et al.  1995^1^ | Erythrocyte lipids (mol%) | **Finland**  Omnivor  Vegan | 11  8 | 11.6 ± 1.1^a^  14.2 ± 1.3^b^ | 13.7 ± 1.0 ^a^  14.3 ± 1.3 ^a^ | 0.1 ± 0.1^a^  0.1 ± 0.1^a^ | 1.4 ± 0.4 ^a^  0.5 ± 0.2 ^b^ | 6.7 ± 0.9^a^  3.3 ± 1.6^b^ |
| Fokkema et al.  2000^1^ | Erythrocyte lipids (mol%) | **Netherlands**  Omnivor  Vegan | 15  12 | 9.78 ± 1.48^a^  11.61 ± 1.67^b^ | 13.76 ± 1.19^a^  14.24 ± 1.09^a^ | 0.17 + 0. 06^a^  0.13 ± 0.11^a^ | 0.55 ± 0.23^a^  0.22 ± 0.20^b^ | 3.90 ± 1.06^a^  2.04 ± 0.87^b^ |
| Kornsteiner et al. 2008^1^ | Erythrocyte phospholipids (mol%) | **Austria**  Omnivor  Flexitarian  Vegetarian  Vegan | 23  13  25  37 | 9.34 ± 1.04^a^  10.67 ± 1.52^b,c^  9.90 ± 0.89^a,b^  11.05 ± 1.46^c^ | 10.11 ± 1.54^a^  9.09 ± 1.58^a,b^  9.76 ± 1.41^a,b^  8.95 ± 1.33^b^ | 0.37 ± 0.25^a^  0.34 ± 0.17 ^a^  0.24 ± 0.16 ^a^  0.28 ± 0.21 ^a^ | 0.35 ± 0.14 ^a^  0.34 ± 0.14 ^a^  0.27 ± 0.10 ^a^  0.16 ± 0.06^b^ | 1.81 ± 0.63^a^  1.84 ± 0.68^a,b^  1.28 ± 0.37^b^  0.87 ± 0.31^c^ |
| Pinto et al.  2019^3^ | Erythrocyte lipids  (% of total fatty acids) | **UK**  Omnivor  Vegan | 24  25 | 11.7 (11.0, 12.3)^a^  13.3 (12.5, 14.1)^b^ | 15.9 (14.9, 16.9)^a^  15.6 (14.4, 16.9)^a^ | 0.34 (0.26, 0.45)^a^  0.32 (0.27, 0.38)^b^ | 1.26 (1.07, 1.45)^a^  0.67 (0.52, 0.81)^b^ | 4.19 (3.63, 4.83)^a^  2.07 (1.85, 2.32)^b^ |
| Chamorro et al. 2020^1^ | Erythrocyte lipids (Mol%) | **Chile**  Omnivor men  Vegan men | 33  34 | 14.8 ± 2.2^a^  16.5 ± 1.9^a^ | 11.9 ± 2.8^a^  10.6 ± 1.8^a^ | 0.12 ± 0.01^a^  0.37 ± 0.1^b^ | 0.32 ± 0.2^a^  0.28 ± 0.3^a^ | 3.44 ± 1.2^a^  1.41 ± 0.6^b^ |
| Chamorro et al.  2023^1^ | Erythrocyte lipids (Mol%) | **Chile**  Omnivor  Pescetarian  Vegan | 35  36  35 | 16.1 ± 2.0^a^  14.3 ± 2.8^a^  16.2 ± 2.6^a^ | 11.2 ± 2.1^a^  10.8 ± 1.5^a^  10.3 ± 1.0^a^ | 1.30 ± 0.05^a^  1.64 ± 0.03^b^  2.28 ± 0.04^c^ | 1.38 ± 0.04^a^  2.02 ± 0.3^b^  1.31 ± 0.05^a^ | 3.12 ± 1.0^a^  4.02 ± 0.9^b^  1.79 ± 0.3^c^ |
| Klein et al. 2025^4^ | Total plasma lipids  (% of total fatty acids) | **Germany**  Omnivor  Flexitarian  Vegetarian  Vegan | 62  69  64  57 | 11.74 / 1.79^a^  12.15 / 2.03^a,b^  12.76 / 2.94^b^  13.84 / 2.40^c^ | 13.98 / 2.38^a^  13.61 / 1.79^a^  13.50 / 1.46^a^  12.93 / 2.66^b^ | 0.16 / 0.07^a^  0.17 / 0.06^a,b^  0.18 / 0.07^a,b^  0.19 / 0.08^b^ | 0.67 / 0.31^a^  0.59 / 0.2^a^  0.49 / 0.26^b^  0.32 / 0.18^c^ | 3.32 / 1.28^a^  3.21 / 1.09^a^  2.66 / 1.04^b^  2.29 / 0.96c |

Values that do not share indices (a,b,c) differ significantly, LA = linoleic acid, ARA = arachidonic acid, ALA = α-linoleinic acid, EPA = eicosapentaenoic acid, DHA = docosahexaenoic acid, ND = not detected

^1^ Data are presented as mean ± standard deviation

^2^ No measure of variation or dispersion was reported

^3^ Data are presented as mean (95% Confidence interval)

^4^ Data are presented as median / Interquartilsrange

^5^ Data are presented as median (25-75 percentile)

**p*-value for the difference between the 4 dietary groups calculated by using ANOVA

References

1. Sanders, T.A.B., F.R. Ellis, and J.W.T. Dickerson, *Studies of vegans: the fatty acid composition of plasma choline phosphoglycerides, erythrocytes, adipose tissue, and breast milk, and some indicators of susceptibility to ischemic heart disease in vegans and omnivore controls12.* The American Journal of Clinical Nutrition, 1978. **31**(5): p. 805-813.

2. Dickerson, J.W.T., T.A.B. Sanders, and F.R. Ellis, *The effects of a vegetarian and vegan diet on plasma and erythrocyte lipids.* Qualitas Plantarum, 1979. **29**(1): p. 85-94.

3. Melchert, H.U., et al., *Fatty acid patterns in triglycerides, diglycerides, free fatty acids, cholesteryl esters and phosphatidylcholine in serum from vegetarians and non-vegetarians.* Atherosclerosis, 1987. **65**(1): p. 159-166.

4. Lee, H.Y., et al., *Serum fatty acid, lipid profile and dietary intake of Hong Kong Chinese omnivores and vegetarians.* European Journal of Clinical Nutrition, 2000. **54**(10): p. 768-773.

5. Fokkema, M.R., et al., *Polyunsaturated fatty acid status of Dutch vegans and omnivores.* Prostaglandins, Leukotrienes and Essential Fatty Acids (PLEFA), 2000. **63**(5): p. 279-285.

6. Rosell, M., et al., *Long-chain n-3 polyunsaturated fatty acids in plasma in British meat-eating, vegetarian, and vegan men.* The American journal of clinical nutrition, 2005. **82**: p. 327-34.

7. Mann, N., et al., *Fatty acid composition of habitual omnivore and vegetarian diets.* Lipids, 2006. **41**(7): p. 637-646.

8. Welch, A.A., et al., *Dietary intake and status of n–3 polyunsaturated fatty acids in a population of fish-eating and non-fish-eating meat-eaters, vegetarians, and vegans and the precursor-product ratio of α-linolenic acid to long-chain n–3 polyunsaturated fatty acids: results from the EPIC-Norfolk cohort.* The American Journal of Clinical Nutrition, 2010. **92**(5): p. 1040-1051.

9. Elorinne, A.L., et al., *Food and Nutrient Intake and Nutritional Status of Finnish Vegans and Non-Vegetarians.* PLoS One, 2016. **11**(2): p. e0148235.

10. Pinto, A.M., et al., *A comparison of heart rate variability, n-3 PUFA status and lipid mediator profile in age- and BMI-matched middle-aged vegans and omnivores.* British Journal of Nutrition, 2017. **117**(5): p. 669-685.

11. Salvador, A.M., et al., *Fatty Acid Profile and Cardiometabolic Markers in Relation with Diet Type and Omega-3 Supplementation in Spanish Vegetarians.* Nutrients, 2019. **11**(7): p. 1659.

12. Chamorro, R., et al., *Diet, Plasma, Erythrocytes, and Spermatozoa Fatty Acid Composition Changes in Young Vegan Men.* Lipids, 2020. **55**(6): p. 639-648.

13. Craddock, J.C., et al., *A Cross-Sectional Comparison of the Whole Blood Fatty Acid Profile and Omega-3 Index of Male Vegan and Omnivorous Endurance Athletes.* Journal of the American Nutrition Association, 2022. **41**(3): p. 333-341.

14. Menzel, J., et al., *Dietary and Plasma Phospholipid Profiles in Vegans and Omnivores—Results from the RBVD Study.* Nutrients, 2022. **14**: p. 2900.

15. Chamorro, R., et al., *Effect of a pescetarian and vegan diet on fatty acid composition in blood and spermatozoa in young healthy men.* Prostaglandins, Leukotrienes and Essential Fatty Acids, 2023. **196**: p. 102582.

16. Gogga, P., et al., *Profiles of Serum Fatty Acids in Healthy Women on Different Types of Vegetarian Diets.* Nutrients, 2024. **16**(4): p. 516.

17. Ågren, J.J., et al., *Fatty acid composition of erythrocyte, platelet, and serum lipids in strict vegans.* Lipids, 1995. **30**(4): p. 365-369.

18. Kornsteiner, M., I. Singer, and I. Elmadfa, *Very Low n–3 Long-Chain Polyunsaturated Fatty Acid Status in Austrian Vegetarians and Vegans.* Annals of Nutrition and Metabolism, 2008. **52**(1): p. 37-47.
